# Biophysical Studies on BEX3, the p75^NTR^-Associated Cell Death Executor, Reveal a High-Order Oligomer with Partially Folded Regions

**DOI:** 10.1371/journal.pone.0137916

**Published:** 2015-09-18

**Authors:** Katia M. S. Cabral, Diana P. Raymundo, Viviane S. Silva, Laura A. G. Sampaio, Laizes Johanson, Luis Fernando Hill, Fabio C. L. Almeida, Yraima Cordeiro, Marcius S. Almeida

**Affiliations:** 1 Instituto de Bioquímica Médica Leopoldo de Meis, Universidade Federal do Rio de Janeiro, Rio de Janeiro, Brazil; 2 Instituto de Biofísica Carlos Chagas Filho, Universidade Federal do Rio de Janeiro, Rio de Janeiro, Brazil; 3 Faculdade de Farmácia, Universidade Federal do Rio de Janeiro, Rio de Janeiro, Brazil; 4 Centro Nacional de Biologia Estrutural e Bioimagem (CENABIO), Universidade Federal do Rio de Janeiro, Rio de Janeiro, Brazil; Weizmann Institute of Science, ISRAEL

## Abstract

BEX3 (Brain Expressed X–linked protein 3) is a member of a mammal-specific placental protein family. Several studies have found the BEX proteins to be associated with neurodegeneration, the cell cycle and cancer. BEX3 has been predicted to be intrinsically disordered and also to represent an intracellular hub for cell signaling. The pro-apoptotic activity of BEX3 in association with a number of additional proteins has been widely supported; however, to the best of our knowledge, very limited data are available on the conformation of any of the members of the BEX family. In this study, we structurally characterized BEX3 using biophysical experimental data. Small angle X-ray scattering and atomic force microscopy revealed that BEX3 forms a specific higher-order oligomer that is consistent with a globular molecule. Solution nuclear magnetic resonance, partial proteinase K digestion, circular dichroism spectroscopy, and fluorescence techniques that were performed on the recombinant protein indicated that the structure of BEX3 is composed of approximately 31% α-helix and 20% β-strand, contains partially folded regions near the N- and C-termini, and a core which is proteolysis-resistant around residues 55–120. The self-oligomerization of BEX3 has been previously reported in cell culture and is consistent with our *in vitro* data.

## Introduction

Growth, differentiation and apoptosis are essential cellular responses, which are regulated by a molecular interaction network that is organized by several regulatory pathways. In addition to being essential to the development of an organism, these regulatory pathways also play a role in disease progression, including cancers and neurodegenerative diseases. For instance, the neurotrophin receptor p75 (p75^NTR^) protein is known to have two contradictory roles in its signaling pathway. It can induce cell cycle arrest followed by apoptosis and can also promote cell survival, which is important for neurite outgrowth [1, 2, 3].

Brain Expressed X–linked protein 3 (BEX3) has been reported to interact with p75^NTR^. Also known as NADE (p75^NTR^–associated cell death executor) [4], BEX3 has been identified as a pro-apoptotic protein [4, 5, 6]. The interaction between p75^NTR^ and BEX3 (UniProt IDs: Q9Z0W1 and Q9WTZ9, respectively) was initially identified by yeast two-hybrid screening and was later confirmed by several well-established *in vitro* and *in vivo* methods [4, 6]. The gene encoding the human homolog of BEX3 (NGFRAP1) is located in the chromosomal region Xq22.1-q22.2; this region is specific to eutherian mammals and contains genes correlated with the adaptive evolution of the neocortex [7].

Immunolocalization studies have indicated that both BEX3 and the p75^NTR^ intracellular domain (p75^NTR^_ICD_) are primarily detected in the cytoplasm, but that they can also move into the cell nucleus [6, 8]. Although the exact role of this nuclear localization is not yet well understood, p75^NTR^_ICD_ has been shown to bind to genomic DNA, which enables it to negatively regulate the transcription of the cyclin E gene [8]. The yeast two-hybrid system identified few other interactors. BEX3 binds to the human hamartin, a tumor suppressor that regulates the mTORC1 (mammalian target of rapamycin complex 1) signaling, DRG-1 (dopamine responsive gene-1), involved in the endosomal multivesicular bodies pathway, and Smac (second mitochondria-derived activator of caspase), a pro-apoptotic factor that activates caspases in the cytochrome c/Apaf-1(apoptotic protease activating factor 1)/caspase-9 pathway, as well as to the NRIF (neurotrophin receptor interacting factor), a transcription regulator involved in p75^NTR^-mediated apoptosis, SC-1 (Schwann cell factor-1), which translocate from the cytoplasm to the nucleus upon NGF binding to p75^NTR^ leading to cell cycle arrest, and 14-3-3ε, the binding partner of a variety of phosphoserine proteins involved in different pathways; these interactions support the pro-apoptotic behavior of this protein [9–13].

Mutagenesis studies have shown that the C-terminus of mouse BEX3 (residues 81 to 124) has no effect on NGF-induced apoptosis in cultured cells, although it can still bind p75^NTR^ [6, 14]. Indeed, this region overlaps with the region that is necessary for BEX3 to interact with 14-3-3ε, Smac, and DRG1 [10–15]. Curiously, the C-terminal regions of human, rat, and mouse BEX3 have functional Rev-like leucine-rich nuclear export signals (LR-NES domain, residues 90 to 100) that are necessary both for self-association and for partner protein interactions [4, 6, 15, 16]. A triple point mutation of conserved hydrophobic residues in the LR-NES motif (L94A, L97A, and L99A) not only confirmed the importance of these residues in nuclear export but also found them to be related to BEX3 self-association and to its interactions with p75^NTR^_DD_, 14-3-3ε, Smac, DRG-1 and hamartin [9–15].

The C-terminus of BEX3 also has two ubiquitination boxes and a C-terminal CaaX motif (CLMP), which are respectively required for targeting BEX3 to the proteasome and mitochondria [4–6, 15]. The two ubiquitination boxes regulate the amount of BEX3 that is present in normal cells [13]. The proteasomal degradation of BEX3 is blocked by its interaction with hamartin [9], whereas it is necessary for BEX3 to associate with mitochondria when they are actively replicating and are therefore primarily perinuclear [17]. Collectively, this information might suggest that BEX3 has an important role during cell growth when oxidative metabolism is elevated [14].

Although there is a substantial amount of data on the activity and function of BEX3, to the best of our knowledge, very little is known about either its 3D structure or the 3D structures of any other member of its family. During the preparation of this manuscript, one report characterizing the BEX family was published [18]. In that report, the authors used circular dichroism to obtain spectra of BEX1 and performed bioinformatics analysis of the entire BEX family; they predicted that BEX proteins have long regions of intrinsic disorder.

Intrinsically disordered proteins (IDPs), which are primarily found in eukaryotes, are non-compact proteins that contain flexible domains and have some degree of predicted secondary or tertiary structure [19–21]. Several unfolded or disordered proteins have been linked to neurodegenerative disease, cardiovascular disease, cancer, and diabetes [22–24]. In fact, BEX3 is known to play a role in apoptosis, and prior studies have already shown that it participates in Zn^2+^-dependent neurodegeneration [25, 26]. A role for this protein in cancer can also be proposed based on its aberrantly high expression in several cancerous cell lines [14].

In this work, we used complementary experimental techniques to characterize the 3D structure of BEX3. Our results revealed that BEX3 exists in solution as a high-order, well-defined oligomer. This oligomer has partially disordered segments at the N- and C-termini and a more compact helical structure at residues 79‒102 that is resistant to proteolysis.

## Materials and Methods

### Recombinant Protein Production

The plasmid pET21d containing the cDNA encoding the mature BEX3 or the death domain of p75^NTR^ (amino acids 326 to 417), with an N-terminal His-tag (MGSSHHHHHHSSGLVPRGSHMATMAENLYFQ), were transformed into the *Escherichia coli* strain BL21 (DE3) or Rosetta (DE3), respectively. Protein expression was achieved by growing the cells in LB (Luria Bertani). The cell culture was shaken at 37°C until an OD_600nm_ of 0.6 was achieved and then induced with 1 mM IPTG. Cells were grown for approximately 3 h at the same temperature and harvested by centrifugation.

The buffer used for purification was 25 mM MES pH 7.0, 150 mM NaCl, and 6 mM β-mercaptoethanol (β-ME), and was named Buffer A. To purify BEX3 using an affinity column, it was necessary to denature the protein with 7 M urea. The cell pellet was resuspended in Buffer A with 7 M urea and complete EDTA-free protease inhibitor cocktail tablets (Sigma-Aldrich Co. LLC). Cells were lysed by sonication in an ice bath, and the debris was removed by centrifugation at 7000 *× g* for 30 minutes; the supernatant was filtered and loaded onto a Ni^2+^ affinity column (HisTrap HP column; GE Healthcare) equilibrated with 90% Buffer A and 10% Buffer B (Buffer A with 500 mM imidazole). A linear gradient of 10–100% Buffer B was used to elute the target protein. The fractions containing BEX3 (determined by SDS-PAGE) were pooled and loaded onto a size exclusion column (16/60 Superdex^TM^ 75, GE Healthcare) equilibrated with Buffer A, in the presence of 7 M urea, and eluted in the same buffer. Fractions containing the recombinant protein BEX3 were concentrated using 10 kDa-cut-off centrifugal filter devices (Millipore). For all of the experiments, the protein was refolded by rapid dilution with Buffer A of the concentrated sample containing urea. Residual urea was removed using a HiTrap desalting column (GE Healthcare). ^13^C- or ^15^N-labeled BEX3 was expressed by growing the cells in M9 minimal medium containing ^15^NH_4_Cl (1 g/L) and (^13^C_6_)-D-glucose (4 g/L) as the sole nitrogen and carbon sources, respectively. Procedures for the expression and purification were similar to those described above. NMR samples were supplemented with 10% D_2_O (v/v) and 3 mM NaN_3_, with urea when indicated. After refolding, BEX3 assumed the same characteristics as the sample before the addition of urea; i.e., it did not bind the Ni^2+^ affinity column, and it eluted as an oligomer on gel filtration chromatography. All experiments carried out in this work were performed at least twice on different days with different set of purified proteins.

For the purification of His-p75^NTR^_DD_, the same protocol was used without urea, and the renaturation step was eliminated. For some experiments with p75^NTR^_DD_, the His-tag was removed by the incubation with TEV protease for 48 hours at 25°C (according to the manufactory Invitrogen™ Life Technology). The identity of purified BEX3 and p75^NTR^_DD_ (with or without His-tag) was confirmed by MALDI-TOF (data not shown).

### Surface Plasmon Resonance (SPR)

p75^NTR^_DD_ (13 μM) was coupled through amine groups to a CM5 chip activated with EDC:NHS (1:1) using a Biacore^®^ X instrument (GE Healthcare) at a flow rate of 10 μL/min until 4,000 response units were coupled. Unreacted sites on the chip were blocked with 1M ethanolamine pH 8.5. Different concentrations of BEX3 (0.2–0.8 μM) was injected in Buffer A at the same flow rate for 3 minutes, and the binding was monitored. For each interaction, the dissociation of BEX3 from p75^NTR^_DD_ was also monitored (~ 3 minutes), and then the sensor chip was regenerated with 1 M NaCl for 30 seconds. In each concentration a double point was checked. Global fits of the series of sensorgrams were performed using BiaEvaluation Software (version 4.1) to calculate *k*_on_, *k*_off_, and *K*_d_^app^ = *k*_off_/*k*_on_.

### Proteinase K (PK) Digestion

PK was used for the partial proteolytic digestion of BEX3. The protein (40 μM BEX3) was diluted in Buffer A (without β-ME) in the absence or presence of 0.5 μg/mL of PK (Sigma-Aldrich, Inc.) at 25°C, for the indicated time in the PK time-course experiments. Each digestion was ended by heating the samples at 95°C, for ten minutes in Laemmli loading buffer. Proteins were separated by 15% SDS-PAGE and stained with colloidal coomassie blue solution. The digestion of 15 μM His-p75^NTR^_DD_ was used as the control for the PK-digestion since it is a well folded domain. Experiments performed without PK were used as negative control, where proteolysis was not detected (data not shown).

### Nuclear Magnetic Resonance (NMR) Spectroscopy

NMR measurements were performed at 13°C on a Bruker Avance III spectrometer operating at ^1^H frequencies of 600 MHz, using the z-axis gradient 5 mm triple resonance cryogenic probe, and of 800 MHz, using the z-axis gradient 5 mm triple resonance probe. ^1^H chemical shifts were referenced to internal sodium-3-(trimethylsilyl)propanesulfonate (DSS). Using the absolute frequency ratios (0.251449530 and 0.101329118), the ^13^C and ^15^N chemical shifts were referenced indirectly to DSS [27, 28]. 2D [^1^H,^15^N]-HSQC, 3D HNCO, 3D HNCACB, 3D CBCA(CO)NH, 3D HBHA(CO)NH, 3D ^15^N-edited (^1^H,^1^H) TOCSY and 3D ^15^N-edited (^1^H,^1^H) NOESY spectra [29] were used to obtain sequence-specific assignments for the polypeptide backbone of 200 μM BEX3 in Buffer A in the presence of 3.6 M urea.

The secondary structure propensity was calculated using the algorithm SSP [30]. The C_∝_, C_β_ and H_∝_ chemical shifts of BEX3 were used as input to calculate the propensity to form α-helix (SSP > 0), or extended conformation such as in β-strands (SSP < 0).

Pulse sequences for the measurement of the longitudinal (*R*_*1*_ = 1/*T*_*1*_) and transverse (*R*_*2*_ = 1/*T*_*2*_) relaxation rates have been described previously [31, 32]. The *R*_*1*_ and *R*_*2*_ relaxation measurements used a series of eight experiments measured at ^1^H frequency of 600 MHz, with relaxation delays ranging from 50 to 1200 ms and 17 to 220 ms, respectively. A recycle delay of 4.0 seconds was used for the *R*_*1*_ relaxation experiments and 1.5 seconds for the *R*_*2*_ experiments. The NMR spectra were processed and analyzed with Topspin 3. The rates *R*_*1*_ and *R*_*2*_ and their standard deviations (S1 Table) were obtained from the fitting as single exponential decays to peak height data, with GraphPad 6.

Steady-state ^15^N{^1^H}-NOEs (S1 Table) were measured at a ^1^H frequency of 600 MHz using TROSY-based experiments [33, 34]. The interscan delay was 5 s, including a saturation period of 3 s. The NMR spectra were processed and analyzed with Topspin 3. The errors in the primary intensity data were taken from the root-mean-square noise of background regions in the spectra [32].

Simulations of the protein dynamics (order parameter *S* ^*2*^) were performed using extended Lipari-Szabo spectral density functions [35]. For simulation we used spectral density that take into consideration three independent motions, fast internal motion in the picosecond timescale, segmental motions in the nanosecond timescale and isotropic overall motion of ~17 ns, which was estimated from the hydrodynamic radius calculated from the gel-filtration retention volume of Bex3 in 3.6 M urea. For simulation we used the magnetic field used in the NMR experiments (B_0_ = 14.09508 T). The parameters that describe the internal dynamics are S^2^_f_ and τ_f_ for the fast picosecond internal motion and S^2^_s_ and τ_s_ for the slow nanosecond segmental motion.

### Circular Dichroism (CD) Spectroscopy

CD experiments were carried out using a Chirascan^TM^ CD Spectrometer (Applied Photophysics) with a 0.1 mm path-length quartz cuvette. CD spectra were recorded using 20 μM BEX3 in 5 times-diluted Buffer A. In this case, the β-ME used was replaced by 0.5 mM DTT to minimize CD signal disturbance. Far-UV spectra were recorded from 190 to 260 nm, averaged over three scans at a speed of 0.5 nm/minutes, and collected in steps of 0.5 nm. The buffer baselines were subtracted from the respective sample spectra. Three measurements were performed for each sample. Secondary structure content was calculated with K2D3 software available at http://k2d3.ogic.ca [36].

### Prediction of Structure from Amino acid Sequence

The prediction of the secondary structure, coiled-coil regions, disordered regions, globular regions, transmembrane helices, and solvent accessibility was performed using the following web servers: PredictProtein (https://www.predictprotein.org) and PsiPred (http://bioinf.cs.ucl.ac.uk/psipred/).

### Mass Spectrometry (MS) Analysis

BEX3 (70 μM) was digested using 0.5 μg/mL PK (1 h at 25°C, pH 7.0) and the digestion products were stopped as mentioned before. PK-resistant fragments were separeted by SDS-PAGE on 18% SDS-PAGE gels, stained with the colloidal coomassie blue solution. Four major PK-resistant bands were excised from the gel and processed for mass spectrometric analysis (Laboratório de Espectrometria de Massas, Laboratório Nacional de Biociências, LNBio).

### Fluorescence Experiments

All of the fluorescence experiments were recorded using a fluorimeter ISS PC-1 (ISS Inc.) at 25°C. Different concentrations of BEX3 were used according to each protocol, as indicated in the figure legends.

The emission spectrum of the tryptophan fluorescence was recorded at wavelengths ranging from 300 to 400 nm using an excitation wavelength of 280 nm. Spectra were collected using 5 or 50 μM BEX3 in the absence or presence of urea.

The compactness of the protein hydrophobic cores was assessed using Bis-ANS (4,4′-dianilino-1,1′-binaphthyl-5,5′-disulfonic acid) fluorescence spectroscopy. The fluorescence emission spectra of Bis-ANS were recorded from 400 to 600 nm using an excitation wavelength of 360 nm, after 5 minutes of incubation of the fluorescent die with BEX3. The dye fluorescence spectroscopy was conducted with 2 μM BEX3 protein plus 6 μM Bis-ANS, with/without 7 M urea. The Bis-ANS stock solution (195 mM) was freshly made in 100% methanol and stored in the dark at 4°C before use. A titration curve was generated to define the Bis-ANS concentration necessary to saturate the binding to BEX3. Spectra of Bis-ANS without protein and protein without Bis-ANS were used as controls.

The fluorescence emission spectra of Thioflavin T (ThT) were recorded from 470 to 520 nm using an excitation wavelength of 450 nm, after 5 minutes of incubation. The ThT fluorescence spectroscopy was conducted with 20 μM BEX3 in the presence of 0.03 μM freshly made dye. The ThT stock solution was prepared in water and filtered with a 0.22 μm syringe filter. The concentration (3.5 M) was estimated by spectrophotometry of ThT diluted in ethanol and using an extinction coefficient of 26,620 M^-1^cm^-1^ at 416 nm [37].

### Small Angle X-ray Scattering (SAXS)

SAXS spectra of BEX3 was acquired at the Brazilian Synchrotron Light Laboratory (LNLS), in São Paulo, Brazil. Scattering curves (I(*q*)) were obtained for 35, 70 and 140 μM BEX3 in Buffer A. Scattering was collected in a mica sample holder using a position-sensitive two-dimension detector (MARCCD), with a wavelength of 0.148 nm, at 25°C. The sample-to-detector distance was set to allow the coverage of a *q* range from 0.008 to 0.208 Ǻ^-1^. Data acquisition was realized with collection of three frames of 300 seconds each for each sample, to control for any damage caused by radiation. The modulus of the scattering value, *q*, was calculated according to the following equation: (*q* = (4π/λ) sinθ), where λ is the employed wavelength and θ the scattering angle. Monodispersity of the samples was confirmed by linear regression of the Guinier domain [38] and by analysis of low *q* values of the scattering curve. The final scattering curves were subtracted from the scattering of the respective buffer and were corrected for the sample’s attenuation. The pair distance distribution function, *P(r)*, of the SAXS data with BEX3 at 140 μM was calculated with the program GNOM, yielding the value of the radius of gyration (Rg) in real space [39].

### Atomic Force Microscopy (AFM)

AFM images were obtained using a Dimension fast scan atomic force microscope (Bruker Corporation) with two different scan modes: Tapping™ and Peak Force Tapping™. For Tapping™, an Icon Head and cantilever AC240TS (240 μm length and 2.5 N/m nominal spring constant) were used, and for Peak Force Tapping™, the Fast Scan Head and FASTSCAN-B cantilever (30 μm length and 2.0 N/m nominal spring constant) were selected. For both imaging methods, 30 μL of 5 μM BEX3 was applied to freshly cleaved mica and allowed to remain in contact for five minutes, followed by two of five-minute washes with 100 μL MilliQ water. The samples were then allowed to dry for 10 minutes.

### Size Exclusion Chromatography (SEC)

We used two SEC columns (SRT SEC-150 and SEC-500 Sepax columns; 7.8×300 mm) to determine the hydrodynamic radius of refolded BEX3 in Buffer A and after overnight incubation in the presence of 3.6 M urea. Columns were run at 1 ml/min and monitored by OD_280 nm_. The following globular proteins were used as standards: thyroglobulin – 86 Å (Sigma-Aldrich, T9145); bovine serum albumin–dimer 45.6 Å and monomer 36.2 Å (Sigma-Aldrich, A7906); carbonic anhydrase – 21.4 Å (Sigma-Aldrich, C7025); cytochrome c – 16.3 Å (Sigma-Aldrich, C2506).

## Results and Discussion

### Recombinant BEX3 is active and form a high order oligomer

**Fig 1:**
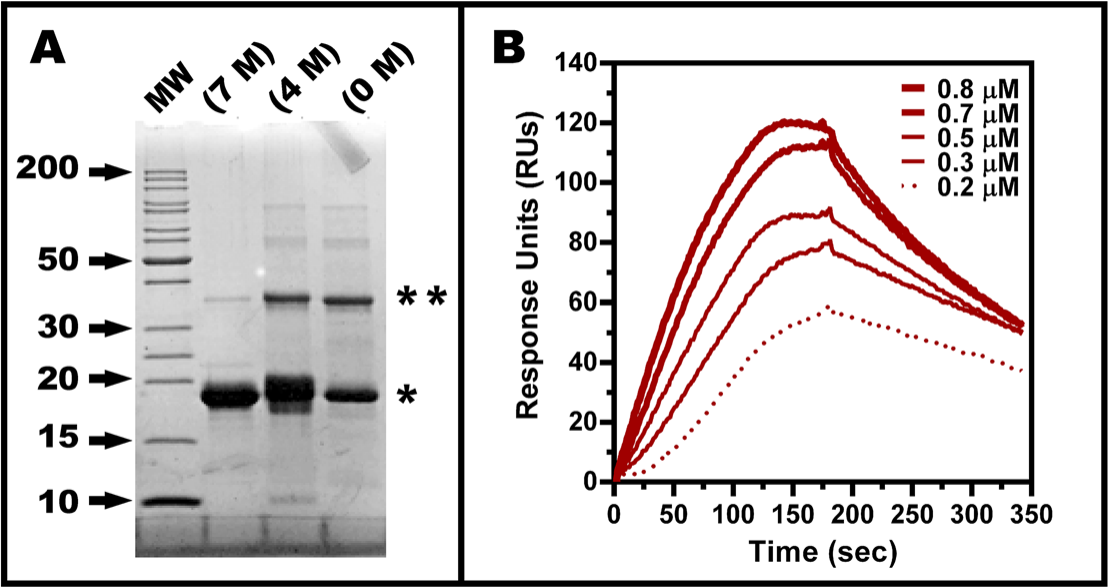
Characterization of BEX3. (A) SDS-PAGE of BEX3 before and after the addition of 4 or 7 M of urea to the sample loading buffer. The molecular weight marker (MW) is shown in kDa for selected proteins. * and ** indicate monomer and dimer of BEX3. (B) Association and dissociation of BEX3 from p75^NTR^_DD_ in surface plasmon resonance studies. Individual sensorgrams were superimposed to show the interactions between BEX3 and p75^NTR^_DD_ at the indicated concentrations. The calculated affinity for the BEX3/p75^NTR^_DD_-interaction was 0.55 μM. The BEX3 sample has been characterized in S1 and S2 Figs.

The recombinant BEX3 protein was efficiently produced in *E*. *coli* and purified as described in “*Materials and Methods*” (S1 Fig). During purification in Superdex 75 with 7 M urea, BEX3 elutes in a peak at 66 mL, corresponding to a globular protein with approximately 53 kDa, and a second peak at 84 mL, which is equivalent to a globular protein with 18 kDa. We have used solely the fractions of the second peak since it has no sign of impurities. It is notable that in the SDS-PAGE shown in Fig 1A, refolded BEX3 derived from this 18 kDa-fraction run at two specific bands, with apparent molecular weight of 19 kDa and 35 kDa; this has already been described elsewhere [4, 15]. Considering the unusual hydrophilic nature of BEX3, we interpret this as an anomalous lower mobility in the SDS-PAGE caused by weaker association to SDS, as described for many other IDPs and the existence of a SDS-resistant oligomer. The SDS-resistant oligomer is susceptible to dissociation in the presence of urea (Fig 1A). Likewise, unusual urea-resistant or SDS-resistant oligomers have been described for the protein α-synuclein [40]. We run the refolded BEX3 on a SEC under non-denaturing conditions (S2 Fig) and identified only a single specie with hydrodynamic radius R_h_ > 86 Å (> 670 kDa).

We tested the association and dissociation between refolded BEX3 and recombinant p75^NTR^_DD_ using SPR binding studies. Sensorgrams showing this interaction confirmed that BEX3 has an active conformation after refolding, as it binds with high affinity (*K*_*d*_^*app*^ = 0.55 μM) to p75^NTR^_DD_ (Fig 1B).

**Fig 2:**
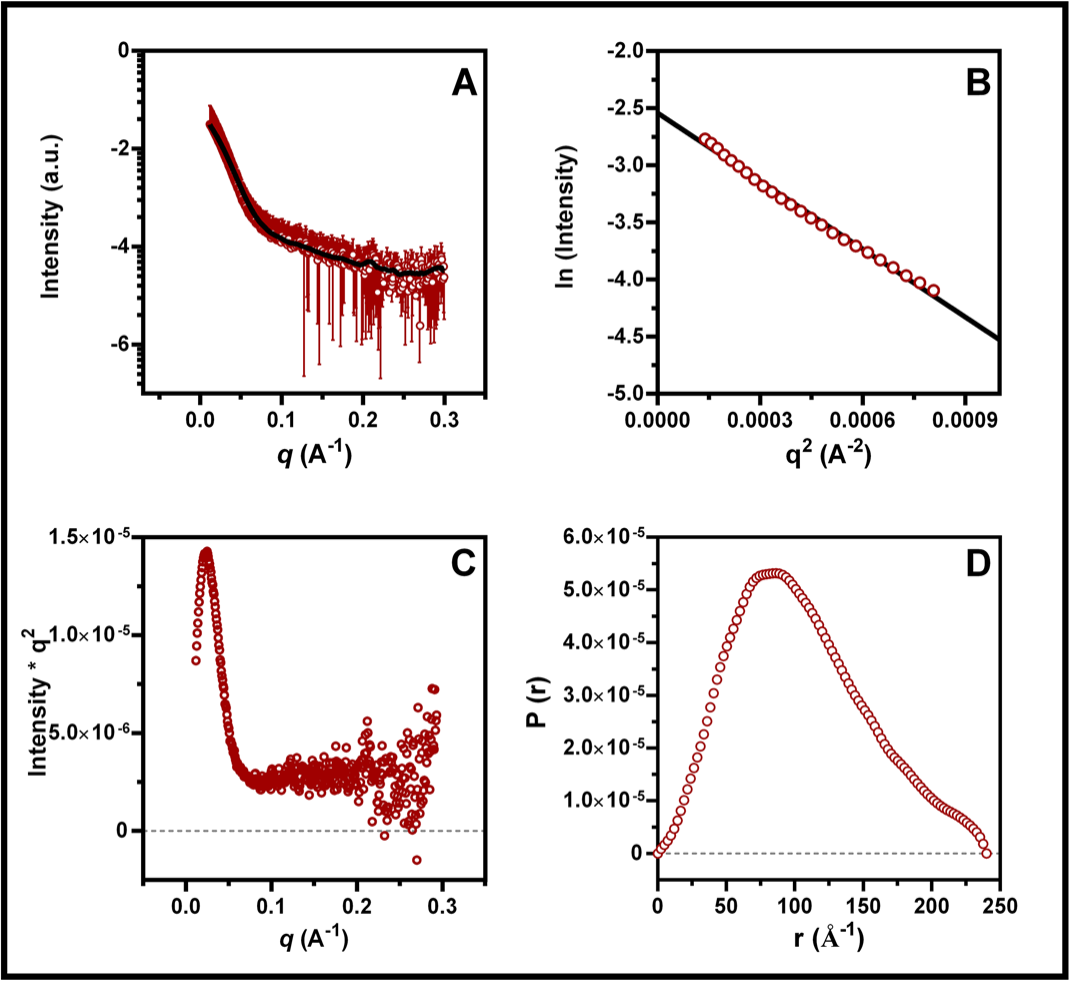
SAXS analysis of BEX3. (A) Experimental scattering curve of BEX3 (70 μM) with the corresponding fit obtained with the program GNOM (solid line). (B) Linear regression of the Guinier domain provided Rg values for BEX3 at 77 Å. (C) Kratky plots of the scattering curves. (D) Pair-distance distribution functions of BEX3 calculated with the program GNOM.

To identify its ability to oligomerize (or even aggregate), we submitted BEX3 to both small-angle X-ray scattering spectroscopy and atomic force microscopy. SAXS experiments can provide valuable information about the overall dimensions of macromolecules in solution at relatively low concentrations [41]. Hence, SAXS data were collected to infer the folding, globularity and shape of BEX3 in solution. The experimentally determined intensity, as a function of the modulus of the scattering vector I(q), and the following GNOM fitting that was performed are shown in Fig 2A. As confirmed by the linear regression of the Guinier plot [42], BEX3 is monodisperse (Fig 2B).

The SAXS curves that were generated at different protein concentrations revealed that BEX3 has no concentration-dependence within the investigated concentration range (S3 Fig). An analysis of the isolated frames did not reveal any damage induced by radiation in the BEX3 sample (data not shown). A Guinier analysis yielded an Rg (radius of gyration) value of 77 Å for BEX3 in the aqueous buffer, which demonstrated that BEX3 is not monomeric under these experimental conditions. Furthermore, by using the GNOM program, it was possible to calculate the pair-distance distribution for the wild type BEX3, which was close to 81 Å (Fig 2D). The P(r) distance data confirmed the Guinier plot analysis and indicated that a higher molecular weight species is present in BEX3 sample.

Using empirical relationships [43] we calculated the hydrodynamic radius of one single chain of BEX3 as 19 Å or 34 Å, considering a globular fold or a denatured protein, respectively. Extrapolating these values to the volume of a sphere ([44]; 29,746 Å^3^, for a globular monomer; 171,792 Å^3^, for a denatured monomer; 2,226,029 Å^3^, for the oligomer), we calculated that the oligomeric specie identified by SAXS can accommodate 13–75 subunits, depending on the compactness of BEX3.

Kratky plots of scattering curves can provide information about the globularity and conformation of a protein in solution [42]. The bell-like shape of the Kratky plot that was based on BEX3 scattering shows that this protein is folded and primarily globular under the tested conditions (Fig 2C). The possibility that BEX3 is a completely unfolded molecule was excluded because, in that case, the values of intensity x q2 (Y-axis) would increase linearly along the q values (X-axis). The P(r) function of BEX3 indicates that this protein has a globular, although slightly elongated, conformation (Fig 2D).

**Fig 3:**
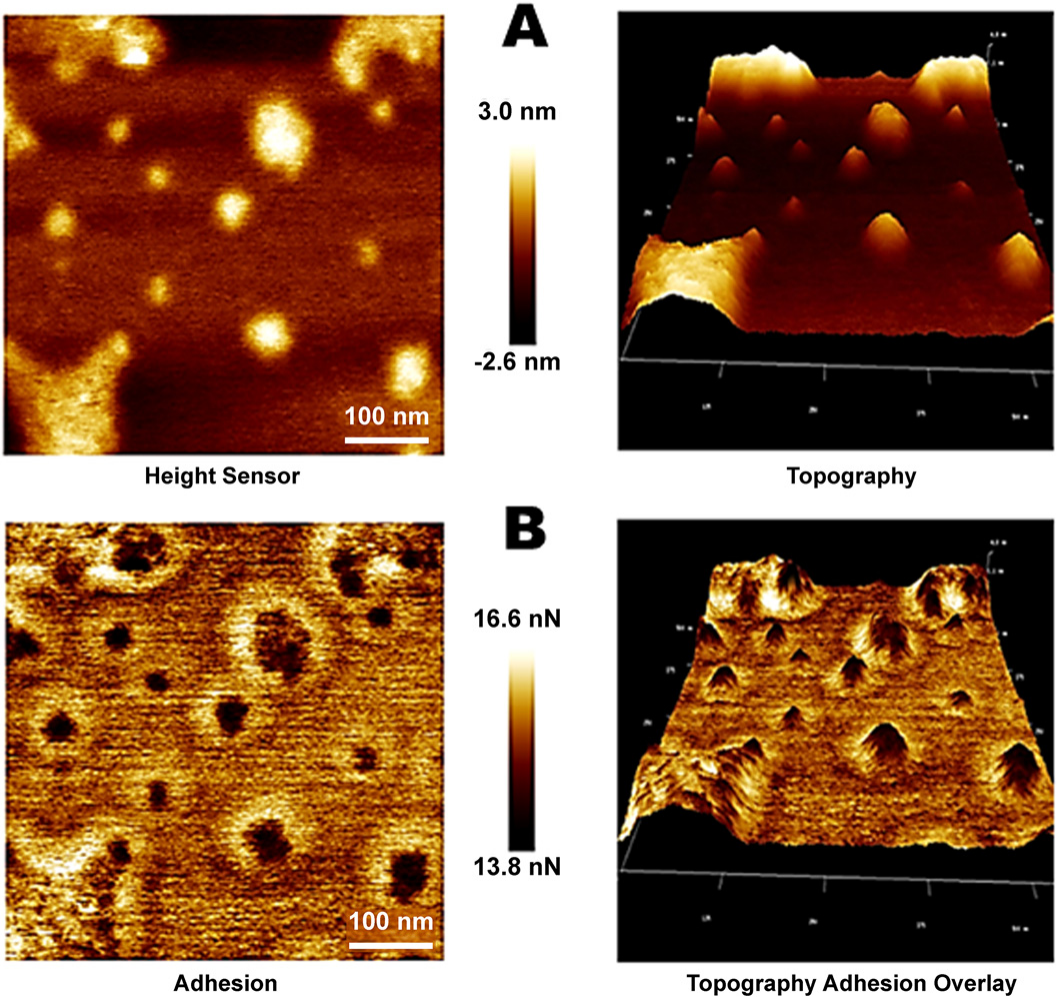
Representative AFM images of BEX3. BEX3 particles were electrostatically adsorbed to mica, as described in *Materials and Methods*. The AFM image was acquired in peak force tapping mode. Topographical images (A) of BEX3 showed recurrent oblate structures. An adhesion map (B) of the corresponding topographical image of BEX3 showed the same structures as having a dense core (darker color) surrounded by a sparse periphery (light color).

Atomic force microscopy (AFM) can provide topographical and mechanical information about a variety of biological complexes, including oligomeric states [45]. Thus, AFM was used to analyze the biophysical properties (adhesion, distribution, and extension) of BEX3. As observed in Fig 3, the AFM images that were acquired in peak force tapping mode show different molecular arrangements for BEX3 on the mica surface. The presence of several variably sized structures confirmed the overall pattern of protein oligomerization (Fig 3). The structures that were observed in the 2D topographical image (Fig 3A, left panel) suggest that BEX3 forms several different complexes, which have an average radius of 125 ± 55 Å and are in either a spherical or oblate ellipsoid arrangement. However, an analysis of the corresponding 2D adhesion map (Fig 3B) shows the presence of a special halo around these complexes and they therefore have a sparser trait. Larger structures, with a variable radius of 375 ± 85 Å, can be observed in Fig 3B. To confirm the size discrepancy that arose between the topography and adhesion images, we applied the more common tapping mode and similar values were obtained between the topography and phase images (S4 Fig). The AFM results corroborated the SAXS and SEC data (Fig 2 and S2 Fig), although the former techniques showed smaller and more homogeneous oligomers in solution. The detection of large oligomers in the AFM experiment may have resulted from an artifact introduced by the deposition of dried BEX3 on the mica surface, though it is interesting to note that all of the oligomers were clearly indicated as having a flexible periphery.

Immunoprecipitation studies have shown that BEX3 is at least dimeric when in cells [15]. Additionally, confocal immunolocalization studies have shown that BEX3 forms intracellular granules (http://www.proteinatlas.org/). These data strongly suggest that the *in vitro* observations that were found in this work can be extrapolated to the intracellular milieu. It is also notable that the purified recombinant BEX3 that was used in this study had an active conformation, as demonstrated by its ability to bind to p75^NTR^_DD_ with high affinity (Fig 1B).

### Circular dichroism analysis reveals secondary structure content in BEX3

**Fig 4:**
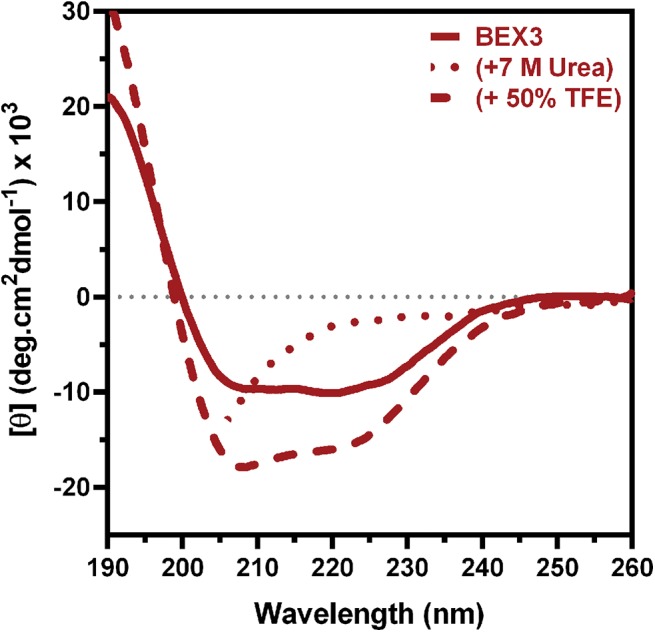
Circular dichroism analysis of BEX3. CD spectra of BEX3 (20 μM) in the absence (solid line) or presence (dashed line) of 50% TFE, and in the presence of 7 M urea (dotted line). Data were fit to a coarse lowess curve using GraphPad Prism v.6.

As observed in Fig 4, BEX3 produced a significant secondary structure signal, with a clear positive band at 190 nm and negative bands near 208 nm and 222 nm (solid line). The deconvolution of this spectrum showed that BEX3 was composed of 31% α-helix and 20% β-strand. However, all of these signals were strengthened in the presence of 50% trifluorethanol (TFE) (dashed line in Fig 4), and they were almost extinguished in the presence of 7 M urea (dotted line in Fig 4). In the presence of 50% TFE, the secondary structure composition that was calculated from spectrum deconvolution included 52% α-helix and 6% β-strand. TFE stabilizes α-helical structures in polypeptide segments that have the propensity to form them, and these results have a significant correlation with the secondary structure that was predicted based on the protein sequence [46]. The ratio of the 222- to the 208-nm peak (*θ*_222_/*θ*_208)_ is used to detect the presence of coiled-coil domains [47, 48]. Indeed, BEX3 has a *θ*_222_/*θ*_208_ ratio of 1.4, which is strongly indicative of the presence of coiled-coil domains. This ratio decreases to 0.9 following the addition of 50% TFE, which is an effect that also been observed when analyzing coiled-coils [48, 49].

### BEX3 has a hydrophobic core

Fluorescence spectroscopy based on tryptophan and Bis-ANS emissions was used to verify the presence of hydrophobic cores in BEX3. It has long been known that the fluorescence emission spectrum of tryptophan is very dependent on the polarity of its surroundings [50]. The fluorescence spectrum shifts to a shorter wavelength, and the intensity of the fluorescence increases as the polarity of the solvent surrounding the tryptophan residue decreases. Typically, the maximum values produced by the fluorescence emission spectra range from 350 nm to 310 nm, respectively, for a highly solvent exposed tryptophan versus a tryptophan that is completely buried within a hydrophobic core (reviewed by [51]). Because of this property, the fluorescence emission spectra produced by tryptophan residues have been widely used to study the hydrophobic cores of proteins.

**Fig 5:**
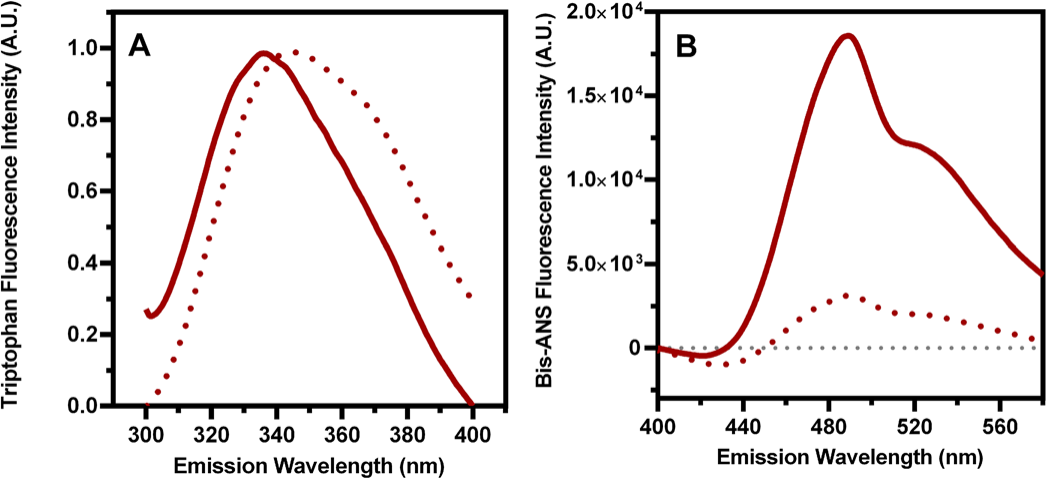
Structure of BEX3 probed by fluorescence. (A) Tryptophan intrinsic emission fluorescence spectra of 50 μM BEX3 were collected at the excitation wavelength of 280 nm. Center of spectral mass is indicated in nm. (B) Bis-ANS emission fluorescence spectra of 2 μM BEX3 were collected at the excitation wavelength of 360 nm. In both panels, the spectra were collected for the proteins in the absence (solid line) or presence (dotted line) of 7 M urea. The data were fit to a coarse lowess curve using GraphPad Prism v.6.

We recorded the tryptophan intrinsic fluorescence spectrum of BEX3 (Fig 5A) and observed a center of spectral mass (CM) near 344 nm (maximum at 336 nm). This emission spectrum suggests that BEX3’s unique tryptophan residue is located in an environment that has intermediate polarity, which can be interpreted as either a hydrophobic core that has some accessibility to the solvent or an uncommon hydrophobic core that includes polar residues. These interpretations are supported by the data that were acquired when the protein was incubated with 7 M urea (dotted lines). In this case, the tryptophan fluorescence showed an increase in the CM to 351 nm (maximum at 345 nm).

In BEX3, the presence of a hydrophobic core that has some accessibility to solvent is further supported by its Bis-ANS fluorescence spectrum (Fig 5B). This probe becomes fluorescent after binding to hydrophobic cores that have significant accessibility to solvent, such as those that are found in molten globule states, oligomers or protein aggregates [52–54].

Notably, the quantum yield of Bis-ANS in the presence of the conformationally intact protein (solid line) was much higher than when the protein was denatured with 7 M urea (dotted line). The spectral intensity of Bis-ANS when it was alone in Buffer A was near zero (dotted line in gray). The strong decrease of Bis-ANS fluorescence signal that was produced with the denatured protein indicates that BEX3 has a partially formed hydrophobic core and supports the idea that BEX3 has a partially folded tertiary structure and/or quaternary structure that is maintained through hydrophobic interactions.

We monitored the denaturation of 50 μM BEX3 with increasing concentrations of Urea (0.5‒7 M) by tryptophan fluorescence and CD (S5 Fig). The denaturation is non-cooperative, typical of non-globular proteins. BEX3 is also moderately more susceptible to denaturation at 1/10 of protein concentration, which is an indication of equilibrium with oligomers.

**Fig 6:**
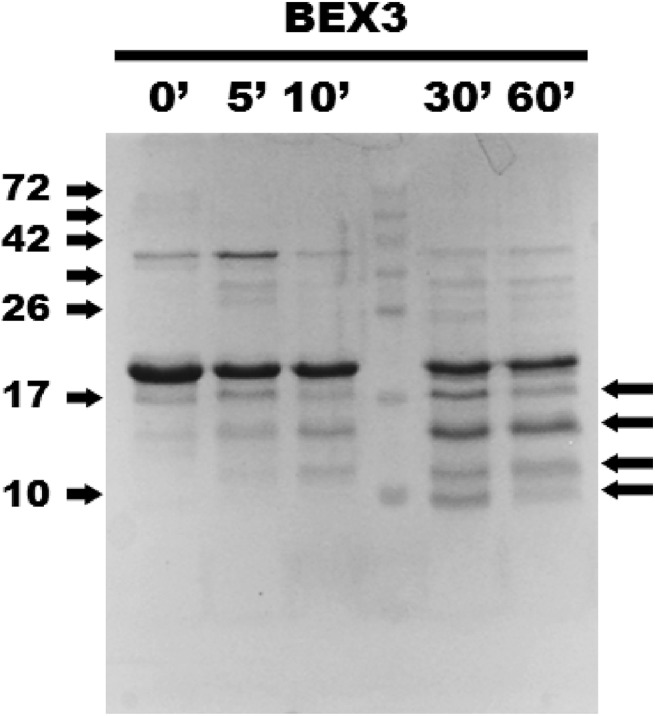
Partial proteolysis of BEX3. 40 μM BEX3 was subjected to partial proteolysis with 0.5 μg/mL PK. A time course of protein fragmentation was detected by SDS-PAGE. The molecular weight marker (MW) is shown in kDa for selected proteins. Arrows on the right show the PK-resistant fragments that were sent for mass spectrometry analysis after 60 minutes of proteolysis.

To further determine the conformational status of the recombinant proteins, we submitted BEX3 and His-p75^NTR^_DD_ to partial Proteinase K (PK) digestion, which is a classical way to identify flexibly disordered regions in proteins [55]. As observed in Fig 6, BEX3 has a notable resistance to proteolysis. Over the time course of the experiment (5–60 minutes), BEX3 was converted into at least four different apparently smaller species (indicated by arrows). These four most abundant PK-resistant peptides that resulted from BEX3 proteolysis (with apparent molecular weights in the range of 5–15 kDa) were excised from a gel, subjected to trypsin and chymotrypsin digestion and identified using LC-MS/MS (as described in *Materials and Methods*). The PK-resistant core encompasses residues 55–120.

The recombinant His-p75^NTR^_DD_ protein was chosen as a control because it has a globular, well-folded C-terminal domain and a flexible 31-amino-acid-long tag at the N-terminus (His-tag). As shown in the S6 Fig, at the very beginning of the digestion, the control protein (His-p75^NTR^_DD_) lost approximately 2.3 kDa (equivalent to the flexibly disordered His-tag) but retained a PK-resistant band of approximately 13.3 kDa (equivalent to the well-folded death domain). After 60 minutes of PK digestion, a 50% abundance of two different PK-resistant species was obtained (bands at 13.3 kDa and 10.4 kDa).

### Intrinsic disorder and residual fold in BEX3

NMR spectroscopy was performed to characterize the 3D structure of BEX3 in more detail. We obtained first-hand experimental evidence of the conformational state of BEX3 by analyzing 1D (^1^H)-NMR spectra (S7 Fig). The separation of the three groups of resonances was evident (black line in S7 Fig): the main chain amide groups were between 7.7 and 8.7 ppm, the aromatic rings between 6.7 and 7.7 ppm, and the aliphatic groups between 0.5 and 4.4 ppm. When using presaturation for water suppression, several backbone amide resonances were lost (gray line in S7 Fig). A similar observation was made with the indolic resonance of BEX3 (10.1 ppm, black line), which disappeared from the presaturation spectrum (gray line). No evidence of β-sheets was observed in the 1D (^1^H) NMR spectra, which is supported by the absence of H_α_ signals near 5 and 6 ppm. The low dispersion of chemical shifts, especially at the main chain amide region and the methyl region (near 0.5–1.0 ppm), together with the susceptibility of the amide moieties to the water presaturation [56], are clearly indicative that this protein has many solvent-exposed H^N^, typical of disordered polypeptide chains [57–61].

**Fig 7:**
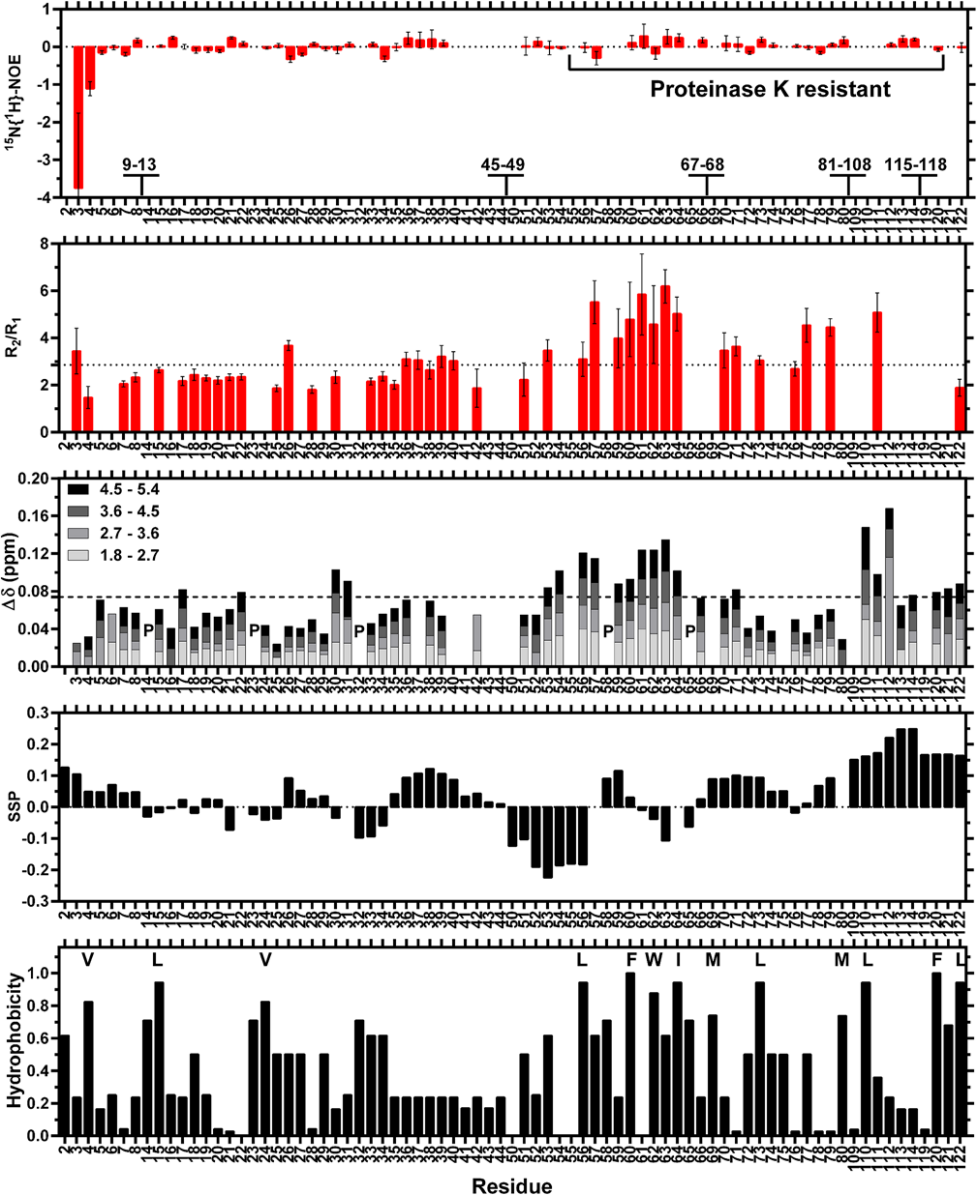
Structural characterization of BEX3 by NMR spectroscopy. Numbers indicate amino acid positions in the BEX3 polypeptide chain. Amino acids that did not have H^N^ assigned are indicated in the upper graph by numbers; P represents prolines. The ^15^N{^1^H}-NOE, *R*_*2*_ */R*_*1*_ and SSP correspond to the protein that was exposed to 3.6 M urea. The Δδ represent the perturbations to chemical shift that were caused by the indicated amounts of urea. ^15^N{^1^H}-NOE values around 0.8 indicate conformational rigidity, whereas negative values represent thermal fluctuations in the conformation. Above-median *R*_*2*_ */R*_*1*_ indicate slower conformational flexibility. Positive values of SSP indicate the presence of α-helices, whereas negative values indicate an extended conformation. The hydrophobicity scale is from Black and Mould, 1991 [69]. The most highly hydrophobic amino acids are indicated by their one-letter codes. The averages and medians are indicated as dashed and dotted lines, respectively.

Triple resonance NMR experiments collected for BEX3 showed very few amide proton (H^N^) signals (data not shown). Higher quality NMR spectra were obtained when BEX3 was partially denatured with 3.6 M urea. According to the chemical denaturation experiments using 0.5‒7 M urea, which were monitored by intrinsic fluorescence and circular dichroism, in the presence of 3.6 M urea BEX3 exits in an equilibrium preserving approximately 33% of its native tertiary structure and 60% of its native secondary structure (S5 Fig). Furthermore, with 3.6 M urea, BEX3 elutes in three peaks (S8 Fig) instead of one (S2 Fig) in the SEC indicating an equilibrium between three discrete species (66.6% with R_h_ = 23.8 Å, 27.7% with R_h_ = 29.9 Å, and 5.6% with R_h_ > 86 Å). It has also been well established that proteins can retain residual 3D structure in denaturing concentrations of urea [62]. Using a set of triple resonance experiments, a total of 64 different amino acid spin systems (out of 117) that had assignable backbone H^N^ were identified in BEX3. The unassigned spin systems included residues 1, 9–13, 45–49 and several residues located in the segment of the protein that is resistant to PK (67, 68, 81–108 and 115–118), as indicated in Fig 7. It is worth noting that these residues must be present in BEX3 according to MALDI-TOF analysis (data not shown). Based on this observation, we believe that many of the 53 spin systems that were missing in the spectra are probably at the interface of a dynamic high-order oligomer (ca. >50 kDa), which is in equilibrium with two smaller BEX3 species. In fact, several peaks with different intensities and line widths were found throughout the entirety of the spectra, which is also indicative of a protein that contains segments that experience different degree of conformational exchange processes.

We have calculated the hydrogen exchange (HX) rate expected in a random coil model of BEX3 using the correction factors for temperature, pH, amino acid neighborhood and empirical rates [63, 64], with the program described by Yu-Zhu Zhang [65], available at https://www.fccc.edu/research/labs/roder/sphere/sphere.html. At pH 7 and 13°C, BEX3 is predicted to have 41 residues with *k*_*ex*_ > 10 s^-1^ (10‒203 s^-1^), which would cause severe reduction in their H^N^ signal. We have identified experimentally the H^N^ of 25 residues from those predicted to have high *k*_*ex*_ (predicted rates of 11.5‒39.5 s^-1^). This indicates that these residues (3, 6, 8, 17, 18, 26, 27, 30, 31, 35–44, 51, 70, 112–114, and 121) does not follow the trend of high *k*_*ex*_ expected for a random coil polypeptide chain, because their H^N^ must be in regions that, at least, significantly experience a conformation that protects them from the solvent. We have tried samples with pH < 7 in order to decrease the exchange rate and favor the detection of the missing spin systems, but BEX3 precipitated.

It is well known that 2D ^15^N{^1^H}-NOE is a very useful method for analyzing the backbone dynamics at pico to nanosecond timescale for individual residues in a protein [32]. Those that undergo fast picosecond motion show NOE with decreased intensity or even opposite sign (minimum intensity at around -3.5). Rigid polypeptide segments show NOE intensity of around 0.8, usually found in secondary structure elements and folded core of proteins. A ^15^N{^1^H}-NOE profile for BEX3 (Fig 7), measured in 3.6 M urea, indicated the existence of flexibly disordered residues in the N-terminal of this protein (ps-ns time scale), which is expected for an IDP.

The median of the *R*_*2*_ */R*_*1*_ data is 2.86, which is typical for the expanded monomeric form of BEX3. Along with the ^15^N{^1^H}-NOE, this data is also a strong indicative of high flexibility for the oligomer of BEX3. Nevertheless we identified few residues with above-median *R*_*2*_ */R*_*1*_, indicating conformational exchange (micro to milliseconds timescale), clearly concentrated around residues 57–64. The augmented *R*_*2*_ */R*_*1*_ was primarily caused by an increase of the *R*_*2*_, which support the existence of conformational exchange processes (S9 Fig).

To better understand the properties of BEX3, we calculated the theoretical values of *R*_*1*_, *R*_*2*_, ^15^N{^1^H}-NOE at 14.1 T [66, 67, 68]. We used the expanded Lipari-Szabo spectral density functions considering three independent motions, a picosecond internal motion, a nanosecond segmental motion and overall isotropic tumbling of the protein. We can meet the observed relaxation parameters taking into consideration the monomeric protein with an isotropic overall tumbling between 7 to 52 ns. A τ_m_ = 7 ns was calculated for fully folded globular BEX3, while a τ_m_ = 52 ns is expected for the hydrated expanded fully unfolded BEX3 [43]. We also used τ_m_ = 17 ns for BEX3 in 3.6 M urea, calculated from the hydrodynamic radius obtained experimentally from the SEC (S8 Fig).

We tried several combination of the parameters of the internal dynamics parameter to describe the experimental observations. In all of them the order parameter (S^2^ = S^2^_f_S^2^_s_) varies from 0.1 to 0.4 and the internal correlation times was in the range of picosecond for the fast component (τ_f_ ~ 100 ps) and the slow segmental component τ_s_ of about 2 ns. As an example, the following condition was able to meet the experimental condition of the median values of NOE = 0 and *R*_*2*_ */R*_*1*_ = 2.86: τ_m_ = 17 ns, *S* ^*2*^ = 0.16; τ_s_ = 1.4 ns and τ_f_ = 100 ps. This is an indication that the relaxation measurements of BEX3 is typical of an IDP, with high disorder and flexibility, even though it still has some degree of order as indicated by the residues in conformational exchange.

The 2D [^15^N,^1^H]-HSQC and 3D HNCO spectra were collected at different concentrations of denaturant (1.8, 2.7 3.6, 4.5 and 5.4 M urea). The spectral dispersion of BEX3 decreased slightly as the concentration of urea increased. For instance, the backbone H^N^ dispersion of the spectrum obtained for the sample that was placed in 1.8 M urea was 0.791 ppm, whereas it was 0.720 ppm for the sample that was placed in 5.4 M urea (data not shown). Indeed, the H^N^ chemical shift variations (Δδ) of BEX3 in different concentrations of denaturant are evident but noticeably small (Fig 7). This is a clear indication that under native conditions, these BEX3 amino acids have a very simple fold, which is similar to the fold that is found under denaturing conditions. Nevertheless, we did identify several amino acids with above average Δδ (dashed line). These residues are concentrated around residues 53–64 and 110–123, and we speculate that these amino acids adopt a relatively more structured fold under native conditions. In fact, these highlighted regions contain the highest concentration of hydrophobic residues [69] among the residues that are shown in Fig 7.

The propensity of BEX3 to form secondary structure was assessed by evaluating the C_α_, C_β_ and H_α_ NMR chemical shifts with the SSP algorithm [30]. It is noteworthy that BEX3 had a tendency to adopt an α-helical conformation remarkably in the C-terminal residues 109–114 and 119–120, and to a lesser extent in residues 2–8, 35–42, and 69–75. On the other hand we detected propensity to form extended conformation in the residues 50–56. No chemical shift data was obtained for the polypeptide segments 9–13, 45–49, 67–68, 81–108 and 115–118, and because of that we were not able to verify the participation of these residues in secondary structures.

The total average secondary structure calculated from the chemical shift assignments of BEX3 in 3.6 M urea is 6.0% of α-helix and 2.7% of β-strands. These values are far below the secondary structure content calculated from the CD spectra of BEX3 in non-denaturing conditions. According to the denaturing curve by urea monitored by CD (S5 Fig), BEX3 contain roughly 60% of its native amount of secondary structure, which can be extrapolated to 10% of α-helix and 4.5% of β-strands, under non-denaturing condition.

This apparent discrepancy between the data obtained from NMR analysis and the CD data (31% α-helix and 20% β-strand; Fig 4) prompted us to hypothesize that BEX3 contains significant amount of secondary structure, within any of the polypeptide segments that were not accessible using NMR spectroscopy, including a long segment (residues 81–108) in the proteinase K-resistant region. A bioinformatic analysis of BEX3 predicted the presence of an α-helix spanning from residues 78 to 102; this secondary structure was further predicted to have a strong tendency to form a coiled-coil.

## Conclusions

To the best of our knowledge, experimental data regarding the conformation of BEX3 were not available prior to this work. Under native solution conditions used in this study, BEX3 was found to exist as a soluble high order oligomer. Oligomers that are observed by AFM have a specific topology that includes diffuse periphery and a compact core. Nevertheless, the self-association of BEX3 has been described in cells [15].

**Fig 8:**
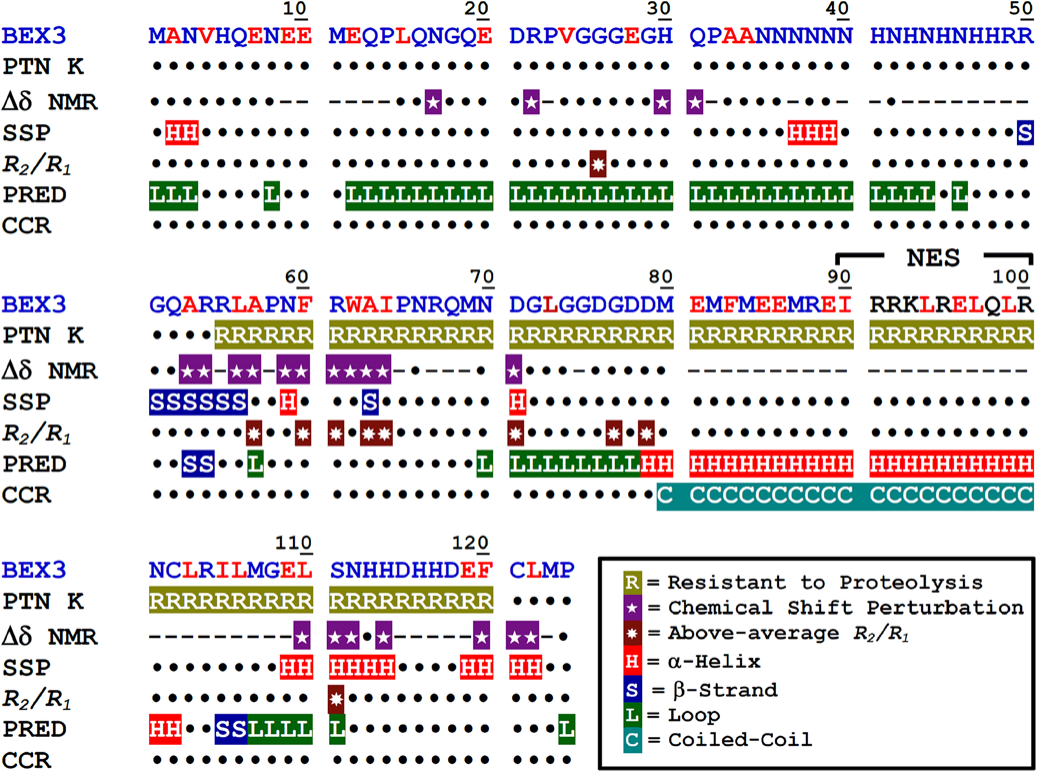
Amino acid sequence of BEX3 highlighting its structural elements. PTN K: amino acid segments resistant to proteinase K. The residues that were predicted to be recognizable by proteinase K by the Peptide Cutter program (http://web.expasy.org/peptide_cutter) are indicated in red. Δδ NMR: represent residues with significant chemical shift perturbation by urea. Dashes represent values that were not determined. SSP: Residues with > 10% secondary structure propensity calculated from C^α^, C^β^ and H^α^ chemical shifts. *R*_*2*_ */R*_*1*_: NMR relaxation indicating residues with below-average conformational flexibility. PRED: Secondary structure prediction from amino acid sequence. CCR: Coiled-coil prediction from amino acid sequence.

According to both the data presented here and the prediction analysis that has already been performed [18], it is reasonable to assume that BEX3 is an IDP. Despite the fact that this molecule has natively unfolded polypeptide segments (i.e., it fails to form a well-defined three-dimensional structure under physiological conditions), the results obtained from our biochemical and biophysical analyses indicated that recombinant BEX3 has regions of ordered 3D structure. A summary of the data obtained by partial proteolysis and NMR that support this hypothesis at residue level is presented with the amino acid sequence of BEX3 and compared to the predicted content of secondary structure (Fig 8).

BEX3 contains a long protease-resistant segment (residues 55–120) that most likely forms a core of buried hydrophobic residues (17 hydrophobic residues out of 25 in BEX3). It is notable that this region contains glutamates that are susceptible to PK (7 glutamates out of 13 in BEX3), but which are somehow not accessible to the protease. NMR analysis indicated that at least residues 53, 54, 56, 57, 59–64, 71 and 110–112 might fold under native conditions. We were not able to identify the H^N^ of residues 81–109 and 115–119 in the NMR spectra, most likely due to extreme line broadening caused by conformational exchange. Under the solution conditions used for the NMR experiments, BEX3 is in an equilibrium of at least two oligomeric species and one monomer; the last being the most abundant specie. This equilibrium can be responsible for the NMR line broadening of the residues at the oligomerization interface. Almost all residues with significantly lowered dynamics as assessed by *R*_*2*_ */R*_*1*_ NMR data are located in the proteinase K-resistant polypeptide segment.

Structural predictions based on the amino acid sequence of BEX3 indicate that the protein has one α-helical polypeptide segment (residues 78–102) that has characteristics of a coiled-coil (Fig 8 and [18]); almost none of these residues are accessible by NMR as mentioned above. The first half of the BEX3 polypeptide chain (residues 1–56) was shown by NMR to have a relatively more flexible conformation. We speculate that at least some portion of this polypeptide segment might form the sparse region that was visualized by AFM (Fig 3).

The CD spectra that were obtained confirmed that BEX3 contains both an α-helix and an extended conformation; they also indicate the formation of a coiled-coil (Fig 4). Denaturation experiments using urea (0.5–7 M) were monitored by tryptophan fluorescence and CD and demonstrated a non-cooperative denaturation curve (S5 Fig), which indicates that BEX3 does not follow the typical denaturation behavior of a protein with a well-defined and compact conformation.

Nevertheless, the differences in the emission fluorescence spectra between native and denatured BEX3 (Fig 4) suggest that this protein has a hydrophobic core that can shield tryptophan 62 from solvent exposure. It is worth noting that this residue lies within a region of the protein that is resistant to proteolysis, undergoes a relatively strong chemical shift perturbation following denaturation with urea and presents lowered dynamics, as indicated by the *R*_*2*_ */R*_*1*_ data.

BEX3 has a nuclear export signal (NES) consensus motif that is located between residues 90 and 100 and is composed of residues L94_,_ L97, and L99; these residues are known to be important both for its self-association and its interactions with binding partners [4, 6, 15]. They are also resistant to proteinase K, which can be correlated to the self-association driven by the NES motif. We speculate that the self-association of BEX3 into a folded core offers a natural mechanism to protect it from degradation and possibly even to regulate its activity, as all known protein-protein interactions that occur between BEX3 and its intracellular effectors (reviewed in [18]) take place at this folded core.

## Supporting Information

S1 Fig BEX3 purification. BEX3 was purified using a two-step procedure, yielding a protein with > 95% purity. (A) HisTrap-Ni^2+^ chromatography. The supernatant of BEX3 cell lysate was loaded onto a 5 mL Ni-affinity column equilibrated with Buffer A (+7 M urea) at a flow rate of 2 ml/min. The protein content was followed by measuring the OD at 280 nm (blue line). The bound BEX3 was eluted with a linear gradient of Imidazole (green line) at a flow rate of 4 mL/min. Fractions (4 mL) were collected (red sticks) during chromatography. (B) Gel filtration chromatography of BEX3 in 7 M urea. Fractions containing BEX3 from the Ni-affinity column were loaded onto a Superdex 75 column (16×600 mm) at a flow rate of 2 mL/min. The gel filtration chromatogram shows the protein content measured by OD_280 nm_ (blue line). The fractions representing pure BEX3 (7–9) were pooled, refolded and then used for the experiments. The inserts show the protein content following each chromatographic step. Each fraction was loaded onto a 15% SDS-PAGE gel and is numbered on the chromatogram. The additional numbers include lane 1 (A), representing the supernatant from the cell lysate before injection onto the column, and lanes 2 (A) and 6 (B), representing the molecular weight marker (broad range, Fermentas).
(TIF)

S2 Fig Size exclusion chromatography (SEC) profile on refolded BEX3. BEX3 or standard globular proteins were loaded onto a SRT SEC-150 and SEC-500 Sepax columns (7.8×300 mm) at a flow rate of 1 mL/min, equilibrated with Buffer A. (A and C) The gel filtration chromatograms show the protein content measured by OD_280 nm_. The elution volumes of globular protein standards are indicated with their hydrodynamic radii (thyroglobulin – 86 Å; bovine serum albumin–dimer 45.6 Å and monomer 36.2 Å; carbonic anhydrase – 21.4 Å; cytochrome c – 16.3 Å). The void (red line) and the maximum inclusion (green line) volumes are indicated (B and D) The elution volumes were plotted against the hydrodynamic radius of each standard protein and fitted to an exponential decay function with base 10.
(TIF)

S3 Fig Scattering intensity of BEX3 at different concentrations as a function of q. (TIF)

S4 Fig AFM surface of BEX3. BEX3 particles were electrostatically adsorbed to mica, as described in the Materials and Methods. The representative AFM image was acquired in Tapping™ mode. (A) Topographical image of the BEX3 oligomeric complex. (B) Phase image of the corresponding topographical image of BEX3.
(TIF)

S5 Fig Effect of urea on the refolded BEX3. (A) The normalized Center of Mass of the intrinsic fluorescence spectra of 5 μM (red) and 50 μM BEX3 (dark red) was measured following overnight treatment with different concentrations of urea, as described in the Materials and Methods. (B) Normalized ellipticity of 50 μM BEX3 measured at 220 nm, following overnight treatment with different concentrations of urea, as described in the Materials and Methods. The extent of BEX3 denaturation with 3.6 M of Urea is indicated in the graphs by dashed lines.
(TIF)

S6 Fig Proteinase K-digestion of His-p75^NTR^DD. A control protein (15 μM His-p75DD) was subjected to 0.3 μg/mL Proteinase K, and fragmentation was detected using 15% SDS-PAGE, as described in the Materials and Methods. Numbered arrows on the left indicate the MW in kDa of selected proteins of based on the molecular weights standard.
(TIF)

S7 Fig 1D [^1^H]-NMR spectra of BEX3. Watergate (black) or H_2_O presaturation (gray) spectra were recorded at 25°C. BEX3 (500 μM) was prepared as described in *Experimental Procedures*. For each experiment, a total of 128 scans with 1.2 s of relaxation delay were collected.
(TIF)

S8 Fig Size exclusion chromatography (SEC) profile on BEX3 with 3.6 M Urea. BEX3 or standard proteins were loaded onto a SRT SEC-150 and SEC-500 Sepax columns (7.8×300 mm) at a flow rate of 1 mL/min, equilibrated with Buffer A containing 3.6 M urea. (A and C) The gel filtration chromatograms show the protein content measured by OD_280 nm_. The elution volumes of globular protein standards are indicated with their hydrodynamic radii (thyroglobulin – 86 Å; bovine serum albumin–dimer 45.6 Å and monomer 36.2 Å; carbonic anhydrase – 21.4 Å; cytochrome c – 16.3 Å). The void (red line) and the maximum inclusion (green line) volumes are indicated (B and D) The elution volumes were plotted against the hydrodynamic radius of each standard protein and fitted to an exponential decay function with base 10.
(TIF)

S9 Fig *R*_*1*_ and *R*_*2*_ data of BEX3. Data collected at 286 K, 600 MHz. The medians are shown as dotted lines. The graphs showing the exponential decay (lines) of the signal intensity for representative residues (colored circles) are shown in function of the delay used for each experiment.
(TIF)

S1 Table NMR relaxation data. Data collected for BEX3 in 3.6 M urea and Buffer A, at 13°C on a Bruker Avance III spectrometer operating at 600 MHz (14.1 T). The standard deviation is shown in the columns identified as SD. The median for each dataset is shown at the bottom. Res# identifies the residue number and NOE stands for heteronuclear ^15^N{^1^H}-NOE.
(DOCX)
