# Supplementary figures and images for: Biophysical Studies on BEX3, the p75NTR-Associated Cell Death Executor, Reveal a High-Order Oligomer with Partially Folded Regions
Source: PLoS One. 2015 Sep 18;10(9):e0137916. doi: 10.1371/journal.pone.0137916 (PMC4575080; doi:10.1371/journal.pone.0137916)

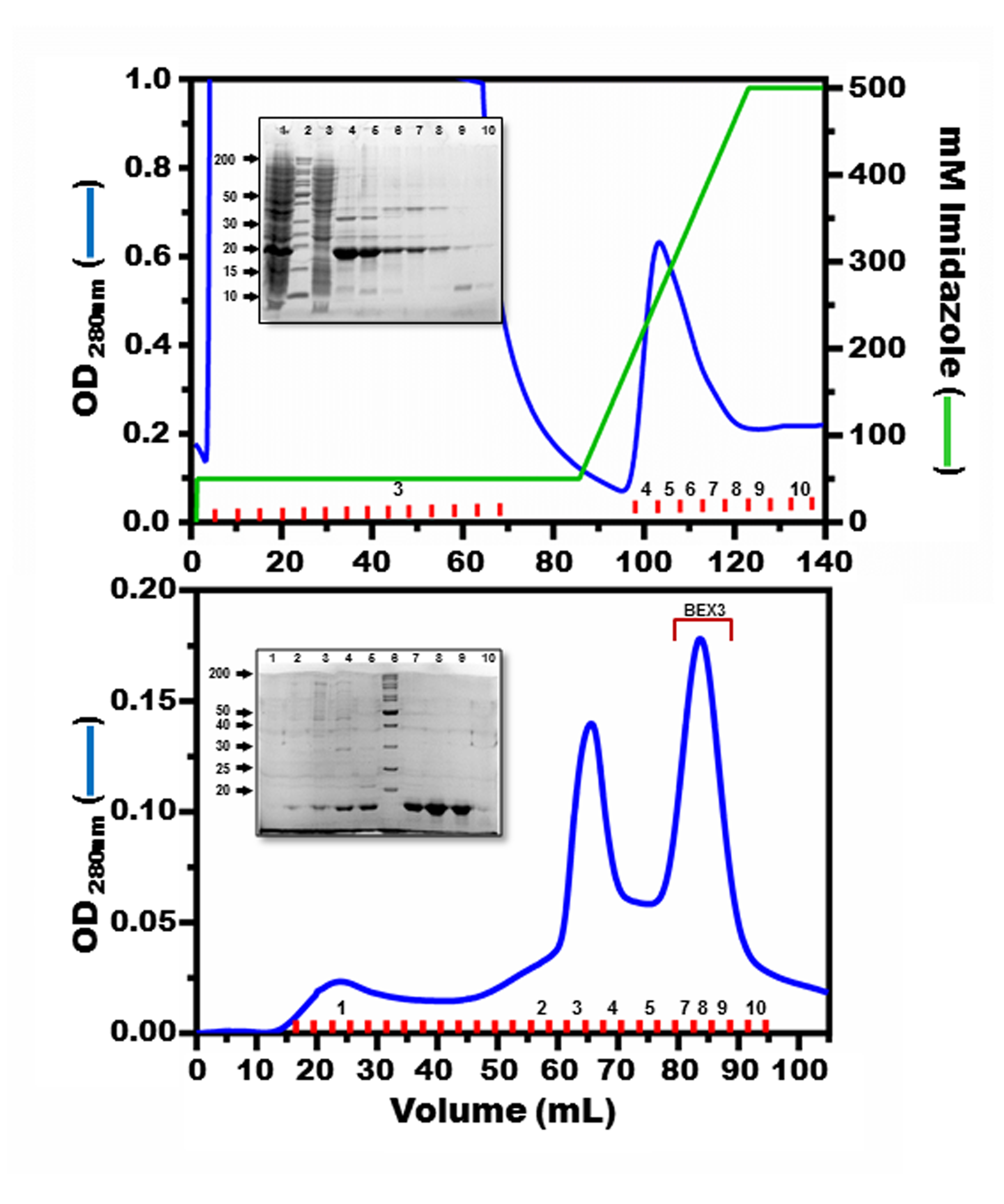

Supplement: S1 Fig — BEX3 was purified using a two-step procedure, yielding a protein with > 95% purity. (A) HisTrap-Ni2+ chromatography. The supernatant of BEX3 cell lysate was loaded onto a 5 mL Ni-affinity column equilibrated with Buffer A (+7 M urea) at a flow rate of 2 ml/min. The protein content was followed by measuring the OD at 280 nm (blue line). The bound BEX3 was eluted with a linear gradient of Imidazole (green line) at a flow rate of 4 mL/min. Fractions (4 mL) were collected (red sticks) during chromatography. (B) Gel filtration chromatography of BEX3 in 7 M urea. Fractions containing BEX3 from the Ni-affinity column were loaded onto a Superdex 75 column (16×600 mm) at a flow rate of 2 mL/min. The gel filtration chromatogram shows the protein content measured by OD280 nm (blue line). The fractions representing pure BEX3 (7–9) were pooled, refolded and then used for the experiments. The inserts show the protein content following each chromatographic step. Each fraction was loaded onto a 15% SDS-PAGE gel and is numbered on the chromatogram. The additional numbers include lane 1 (A), representing the supernatant from the cell lysate before injection onto the column, and lanes 2 (A) and 6 (B), representing the molecular weight marker (broad range, Fermentas). (TIF) [file pone.0137916.s001.tif]

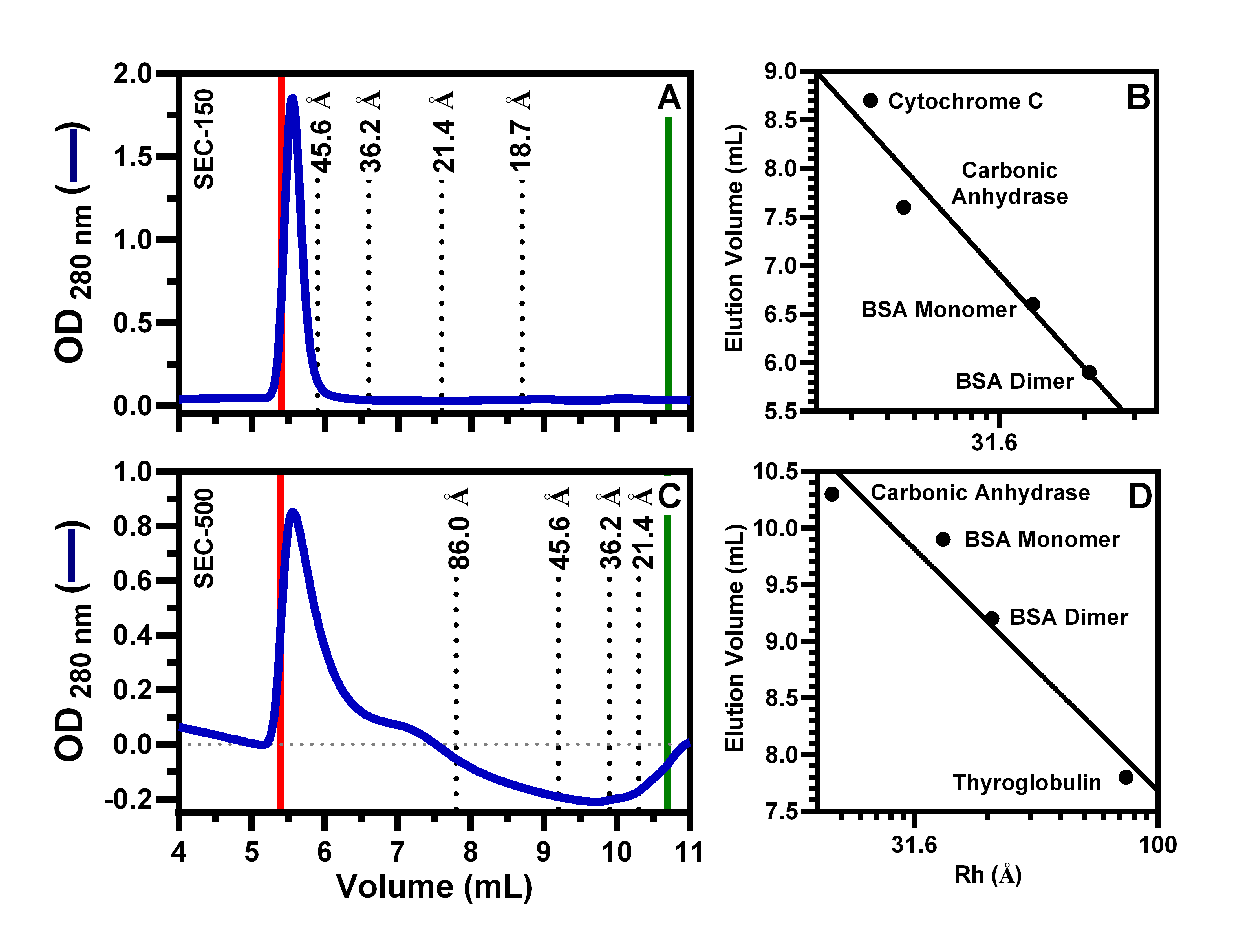

Supplement: S2 Fig — BEX3 or standard globular proteins were loaded onto a SRT SEC-150 and SEC-500 Sepax columns (7.8×300 mm) at a flow rate of 1 mL/min, equilibrated with Buffer A. (A and C) The gel filtration chromatograms show the protein content measured by OD280 nm. The elution volumes of globular protein standards are indicated with their hydrodynamic radii (thyroglobulin – 86 Å; bovine serum albumin–dimer 45.6 Å and monomer 36.2 Å; carbonic anhydrase – 21.4 Å; cytochrome c – 16.3 Å). The void (red line) and the maximum inclusion (green line) volumes are indicated (B and D) The elution volumes were plotted against the hydrodynamic radius of each standard protein and fitted to an exponential decay function with base 10. (TIF) [file pone.0137916.s002.tif]

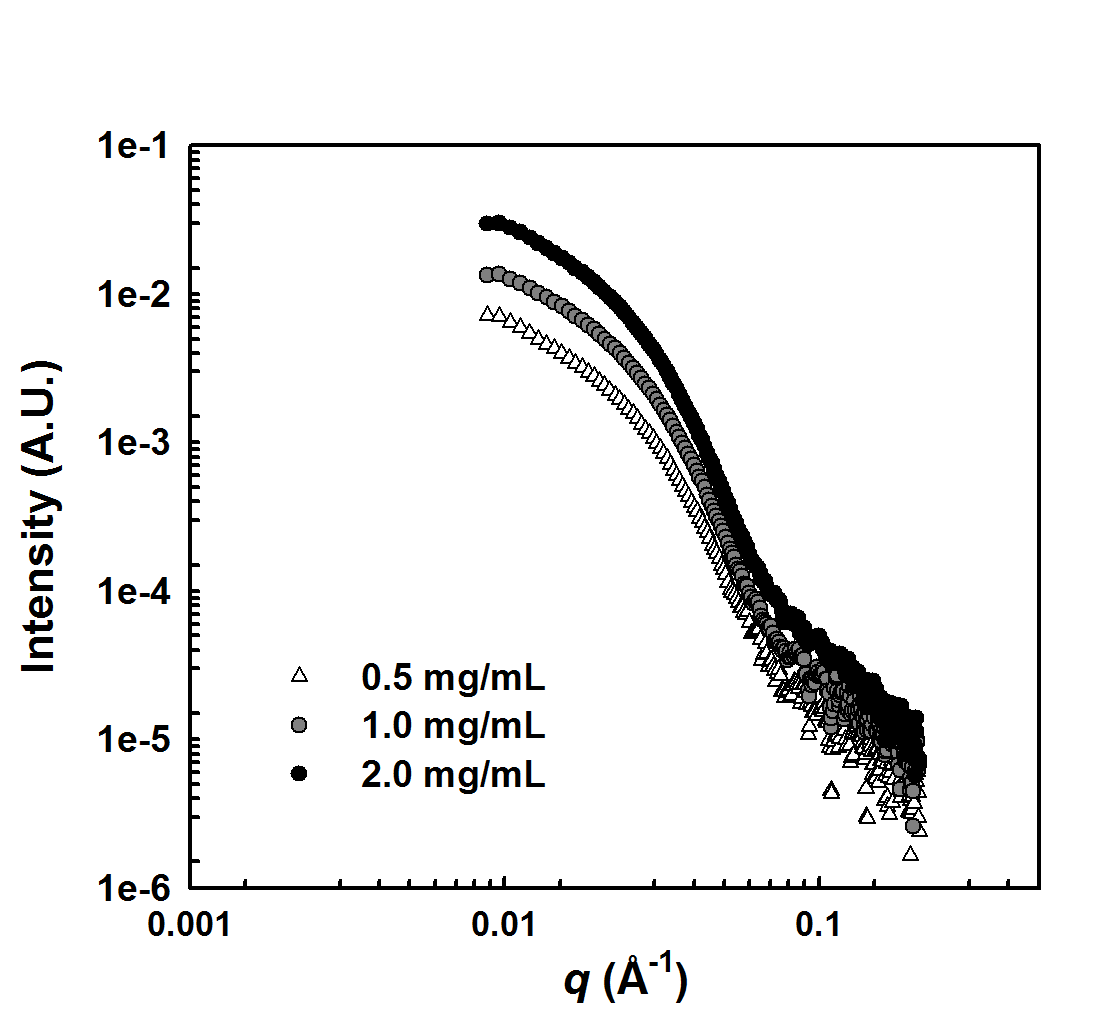

Supplement: S3 Fig — (TIF) [file pone.0137916.s003.TIF]

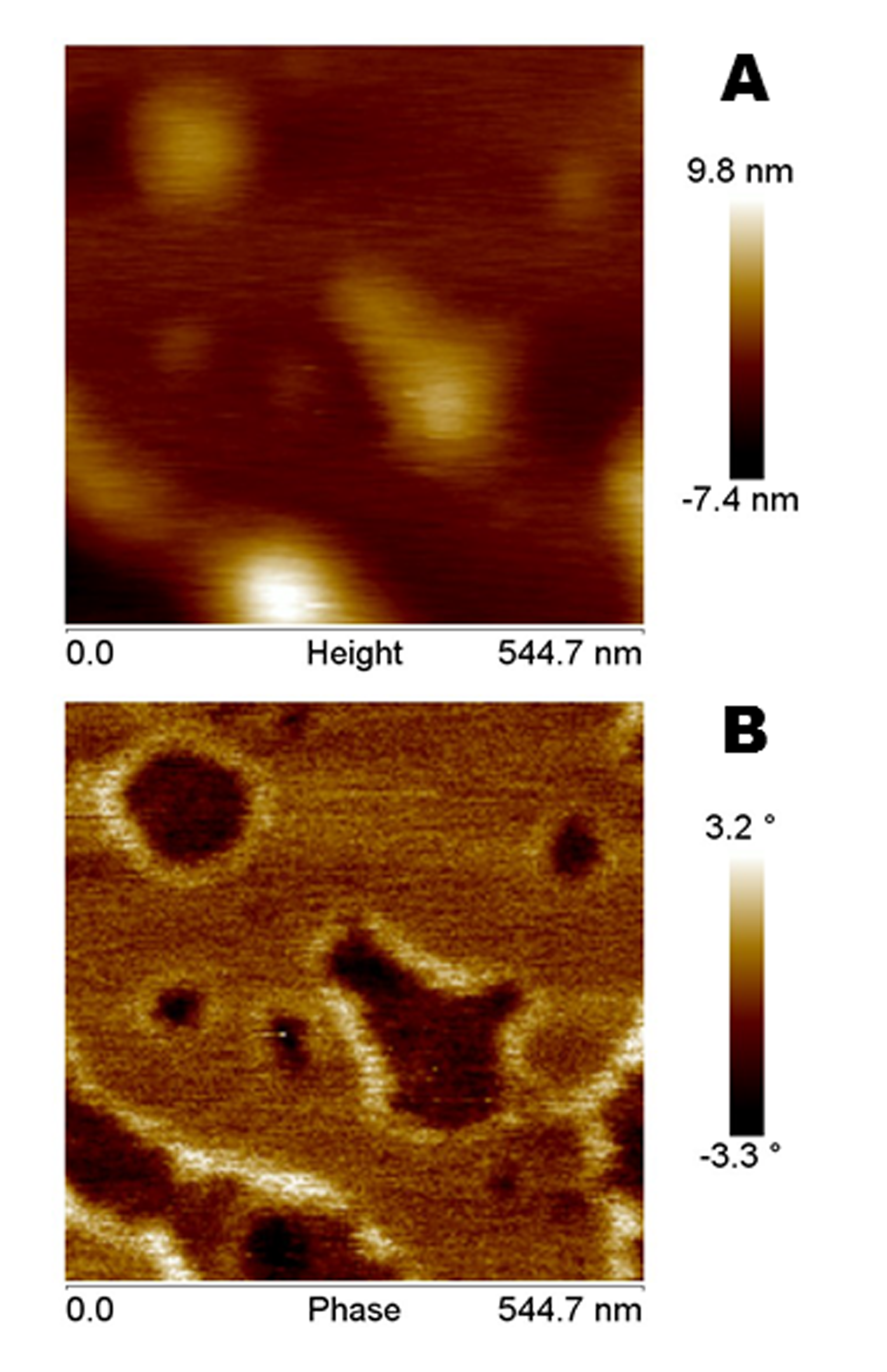

Supplement: S4 Fig — BEX3 particles were electrostatically adsorbed to mica, as described in the Materials and Methods. The representative AFM image was acquired in Tapping™ mode. (A) Topographical image of the BEX3 oligomeric complex. (B) Phase image of the corresponding topographical image of BEX3. (TIF) [file pone.0137916.s004.tif]

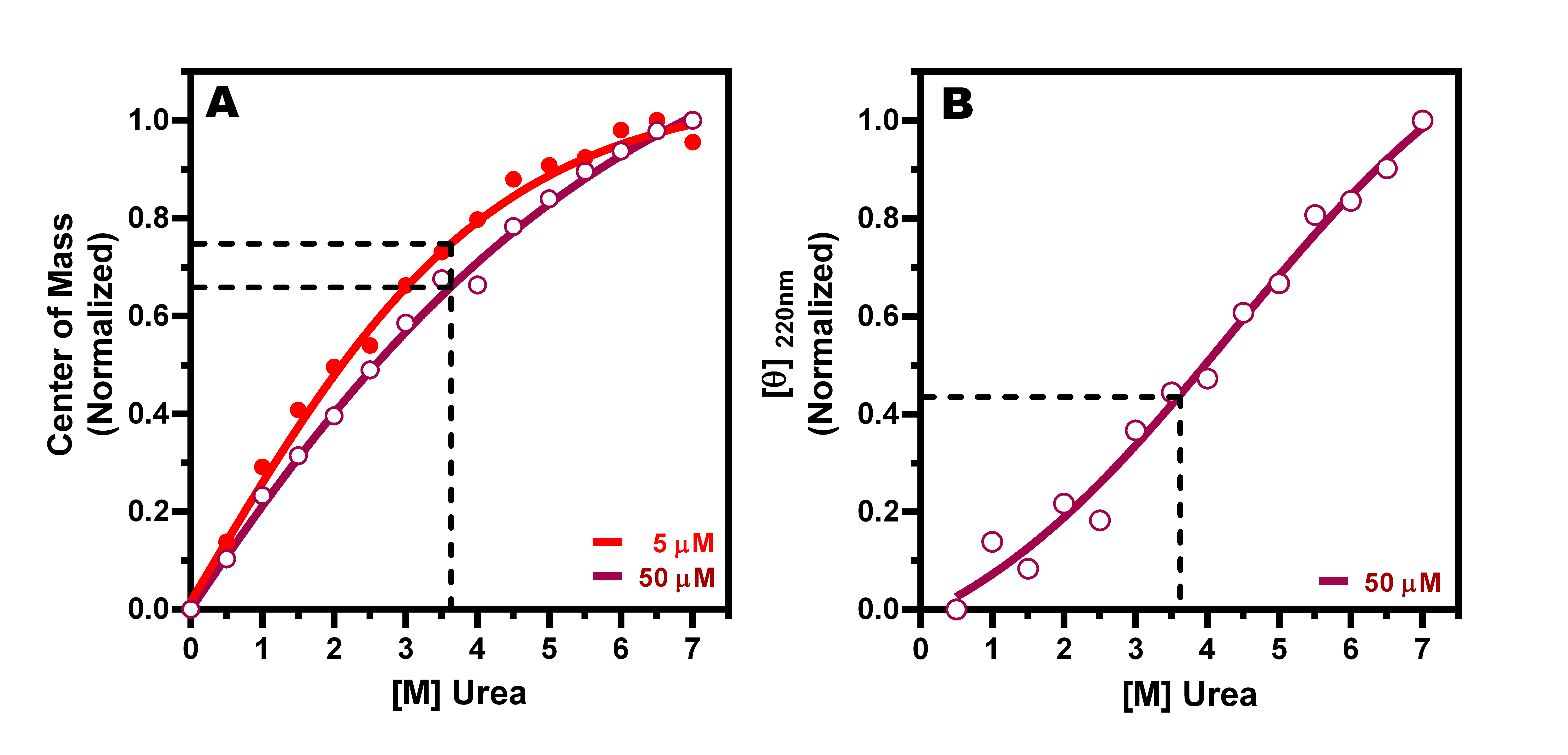

Supplement: S5 Fig — (A) The normalized Center of Mass of the intrinsic fluorescence spectra of 5 μM (red) and 50 μM BEX3 (dark red) was measured following overnight treatment with different concentrations of urea, as described in the Materials and Methods. (B) Normalized ellipticity of 50 μM BEX3 measured at 220 nm, following overnight treatment with different concentrations of urea, as described in the Materials and Methods. The extent of BEX3 denaturation with 3.6 M of Urea is indicated in the graphs by dashed lines. (TIF) [file pone.0137916.s005.tif]

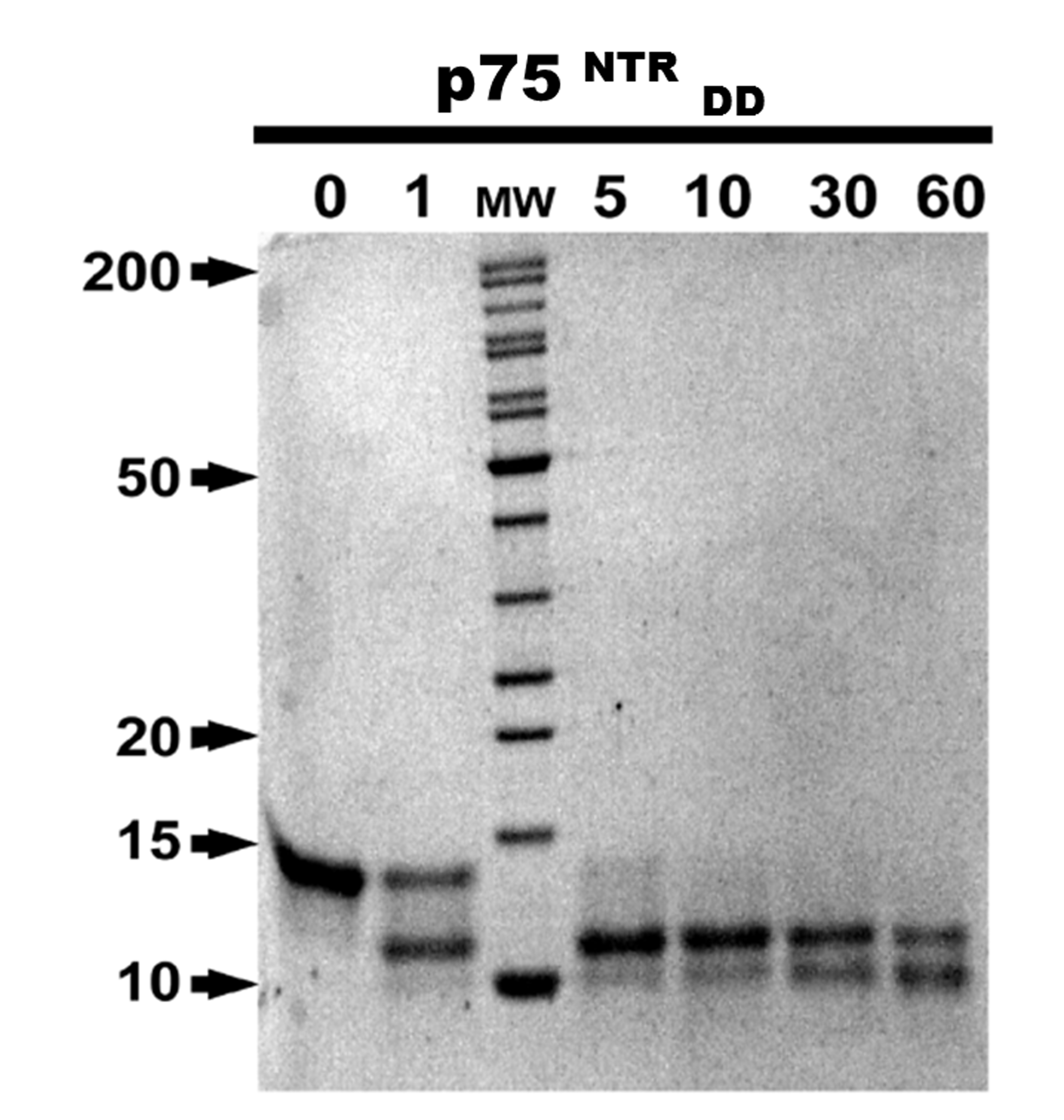

Supplement: S6 Fig — A control protein (15 μM His-p75DD) was subjected to 0.3 μg/mL Proteinase K, and fragmentation was detected using 15% SDS-PAGE, as described in the Materials and Methods. Numbered arrows on the left indicate the MW in kDa of selected proteins of based on the molecular weights standard. (TIF) [file pone.0137916.s006.tif]

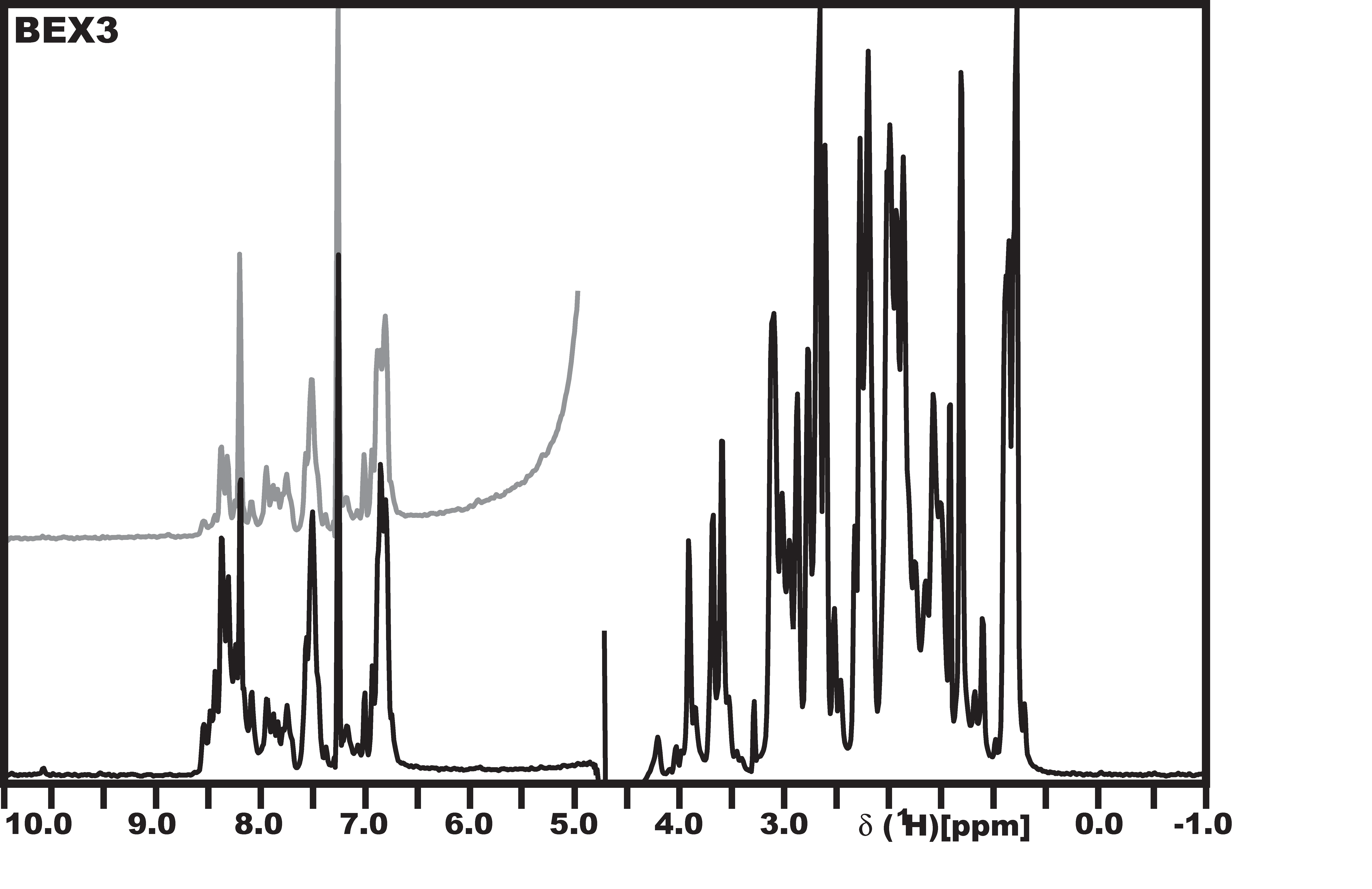

Supplement: S7 Fig — Watergate (black) or H2O presaturation (gray) spectra were recorded at 25°C. BEX3 (500 μM) was prepared as described in Experimental Procedures. For each experiment, a total of 128 scans with 1.2 s of relaxation delay were collected. (TIF) [file pone.0137916.s007.tif]

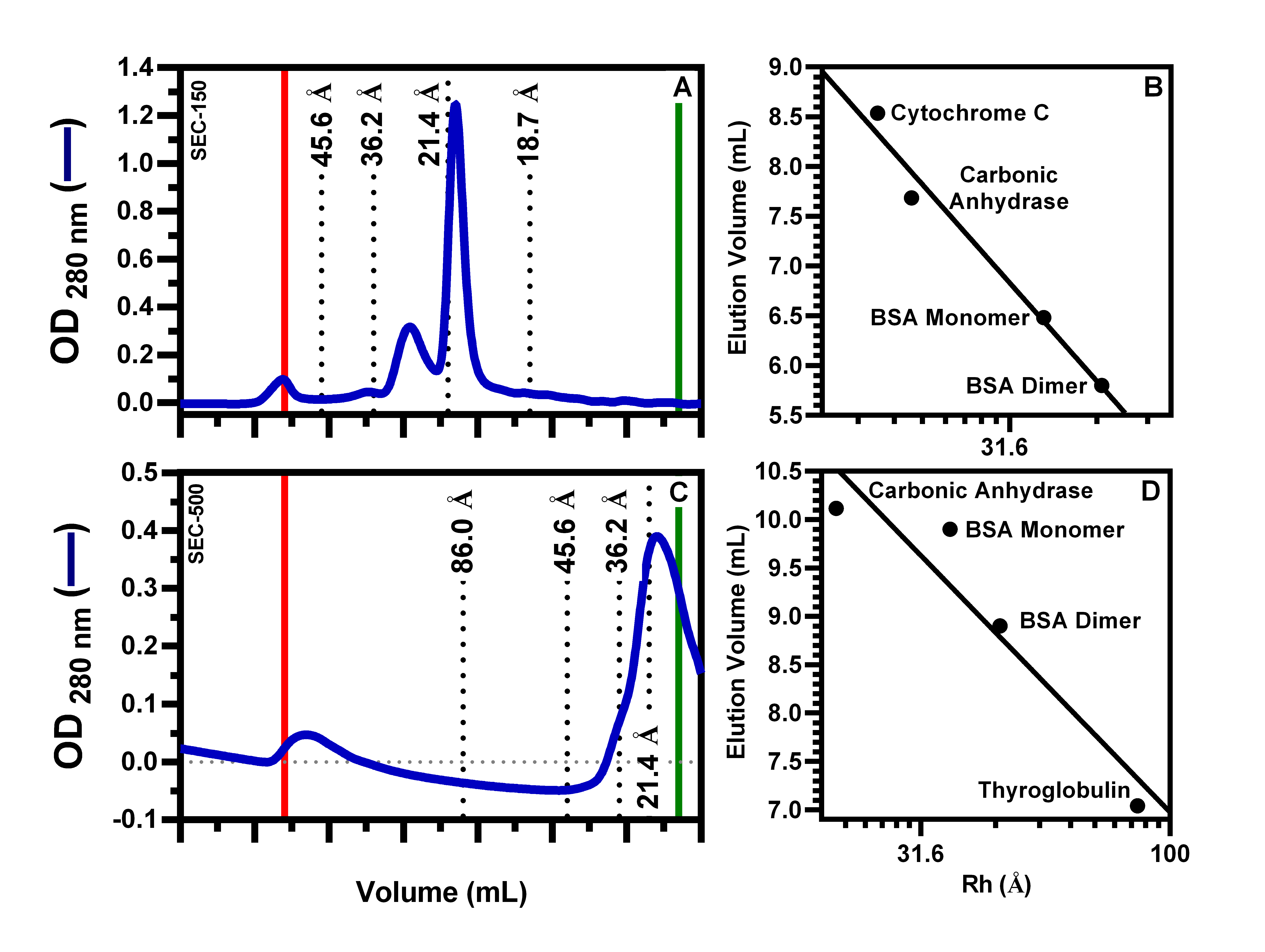

Supplement: S8 Fig — BEX3 or standard proteins were loaded onto a SRT SEC-150 and SEC-500 Sepax columns (7.8×300 mm) at a flow rate of 1 mL/min, equilibrated with Buffer A containing 3.6 M urea. (A and C) The gel filtration chromatograms show the protein content measured by OD280 nm. The elution volumes of globular protein standards are indicated with their hydrodynamic radii (thyroglobulin – 86 Å; bovine serum albumin–dimer 45.6 Å and monomer 36.2 Å; carbonic anhydrase – 21.4 Å; cytochrome c – 16.3 Å). The void (red line) and the maximum inclusion (green line) volumes are indicated (B and D) The elution volumes were plotted against the hydrodynamic radius of each standard protein and fitted to an exponential decay function with base 10. (TIF) [file pone.0137916.s008.tif]

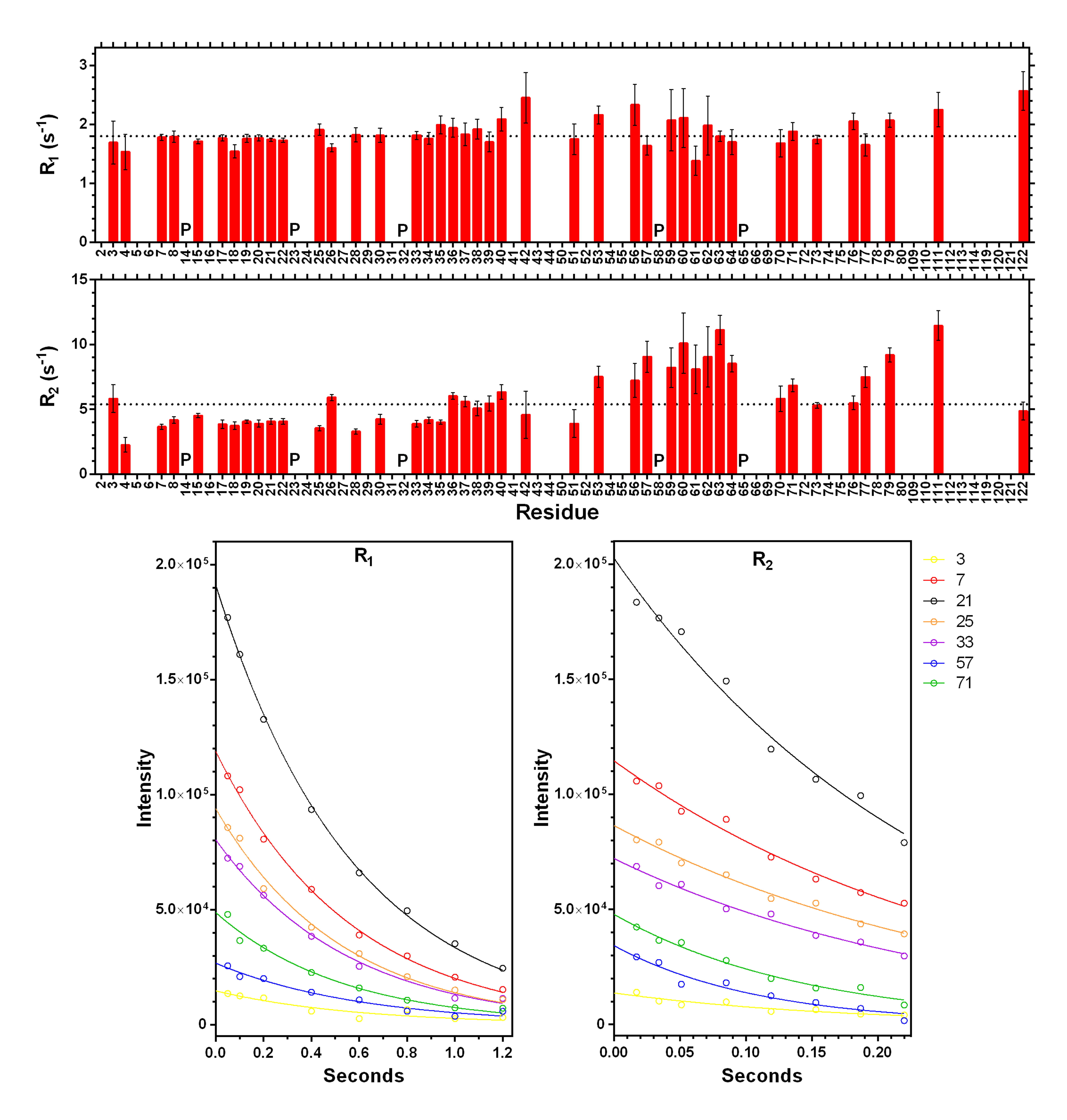

Supplement: S9 Fig — Data collected at 286 K, 600 MHz. The medians are shown as dotted lines. The graphs showing the exponential decay (lines) of the signal intensity for representative residues (colored circles) are shown in function of the delay used for each experiment. (TIF) [file pone.0137916.s009.tif]
