# Supplementary material for: Biophysical Studies on BEX3, the p75NTR-Associated Cell Death Executor, Reveal a High-Order Oligomer with Partially Folded Regions
Source: PLoS One. 2015 Sep 18;10(9):e0137916. doi: 10.1371/journal.pone.0137916 (PMC4575080; doi:10.1371/journal.pone.0137916)
Supplement: S1 Table — Data collected for BEX3 in 3.6 M urea and Buffer A, at 13°C on a Bruker Avance III spectrometer operating at 600 MHz (14.1 T). The standard deviation is shown in the columns identified as SD. The median for each dataset is shown at the bottom. Res# identifies the residue number and NOE stands for heteronuclear 15N{1H}-NOE. (DOCX) [file pone.0137916.s010.docx]

| **S1 Table: NMR relaxation data.** | | | | | | | | |
| --- | --- | --- | --- | --- | --- | --- | --- | --- |
| **Res#** | **NOE** | **SD** | ***R_2_*** | **SD** | ***R_1_*** | **SD** | ***R_2_/R_1_*** | **SD** |
| 2 |  |  |  |  |  |  |  |  |
| 3 | -3.75 | 1.99 | 5.83 | 1.07 | 1.69 | 0.36 | 3.44 | 0.97 |
| 4 | -1.11 | 0.19 | 2.25 | 0.57 | 1.53 | 0.30 | 1.47 | 0.47 |
| 5 | -0.15 | 0.05 |  |  |  |  |  |  |
| 6 | -0.02 | 0.06 |  |  |  |  |  |  |
| 7 | -0.20 | 0.05 | 3.65 | 0.21 | 1.78 | 0.05 | 2.05 | 0.13 |
| 8 | 0.17 | 0.06 | 4.18 | 0.24 | 1.79 | 0.10 | 2.33 | 0.19 |
| 14 |  |  |  |  |  |  |  |  |
| 15 | 0.02 | 0.03 | 4.51 | 0.17 | 1.71 | 0.04 | 2.64 | 0.11 |
| 16 | 0.24 | 0.04 |  |  |  |  |  |  |
| 17 | 0.00 | 0.06 | 3.84 | 0.33 | 1.77 | 0.05 | 2.17 | 0.19 |
| 18 | -0.10 | 0.07 | 3.74 | 0.27 | 1.54 | 0.11 | 2.44 | 0.24 |
| 19 | -0.09 | 0.06 | 4.05 | 0.14 | 1.76 | 0.07 | 2.30 | 0.12 |
| 20 | -0.12 | 0.04 | 3.90 | 0.26 | 1.77 | 0.05 | 2.20 | 0.16 |
| 21 | 0.24 | 0.03 | 4.06 | 0.23 | 1.74 | 0.03 | 2.33 | 0.14 |
| 22 | 0.09 | 0.05 | 4.06 | 0.21 | 1.73 | 0.04 | 2.35 | 0.13 |
| 23 |  |  |  |  |  |  |  |  |
| 24 | -0.03 | 0.03 |  |  |  |  |  |  |
| 25 | 0.03 | 0.06 | 3.54 | 0.19 | 1.91 | 0.10 | 1.86 | 0.14 |
| 26 | -0.33 | 0.08 | 5.91 | 0.23 | 1.60 | 0.07 | 3.68 | 0.21 |
| 27 | -0.21 | 0.04 |  |  |  |  |  |  |
| 28 | 0.08 | 0.04 | 3.29 | 0.20 | 1.82 | 0.12 | 1.81 | 0.16 |
| 29 | -0.05 | 0.05 |  |  |  |  |  |  |
| 30 | -0.08 | 0.10 | 4.24 | 0.38 | 1.81 | 0.12 | 2.34 | 0.26 |
| 31 | 0.06 | 0.06 |  |  |  |  |  |  |
| 32 |  |  |  |  |  |  |  |  |
| 33 | 0.07 | 0.05 | 3.88 | 0.24 | 1.81 | 0.07 | 2.15 | 0.15 |
| 34 | -0.32 | 0.07 | 4.16 | 0.25 | 1.76 | 0.10 | 2.37 | 0.20 |
| 35 | -0.01 | 0.10 | 4.00 | 0.17 | 1.99 | 0.15 | 2.01 | 0.18 |
| 36 | 0.23 | 0.16 | 6.03 | 0.27 | 1.94 | 0.16 | 3.10 | 0.29 |
| 37 | 0.18 | 0.21 | 5.59 | 0.40 | 1.83 | 0.19 | 3.06 | 0.39 |
| 38 | 0.20 | 0.25 | 5.07 | 0.56 | 1.92 | 0.17 | 2.64 | 0.38 |
| 39 | 0.10 | 0.08 | 5.46 | 0.59 | 1.70 | 0.17 | 3.21 | 0.47 |
| 40 |  |  | 6.33 | 0.56 | 2.09 | 0.20 | 3.03 | 0.39 |
| 41 |  |  |  |  |  |  |  |  |
| 42 |  |  | 4.57 | 1.82 | 2.45 | 0.43 | 1.87 | 0.81 |
| 43 |  |  |  |  |  |  |  |  |
| 44 |  |  |  |  |  |  |  |  |
| 50 |  |  |  |  |  |  |  |  |
| 51 | 0.02 | 0.24 | 3.89 | 1.08 | 1.75 | 0.26 | 2.23 | 0.70 |
| 52 | 0.14 | 0.11 |  |  |  |  |  |  |
| 53 | -0.03 | 0.18 | 7.50 | 0.81 | 2.16 | 0.15 | 3.47 | 0.45 |
| 54 | -0.03 | 0.03 |  |  |  |  |  |  |
| 55 |  |  |  |  |  |  |  |  |
| 56 | -0.02 | 0.13 | 7.22 | 1.31 | 2.33 | 0.35 | 3.10 | 0.73 |
| 57 | -0.30 | 0.18 | 9.06 | 1.20 | 1.64 | 0.16 | 5.52 | 0.91 |
| 58 |  |  |  |  |  |  |  |  |
| 59 |  |  | 8.22 | 1.53 | 2.07 | 0.52 | 3.98 | 1.25 |
| 60 | 0.11 | 0.19 | 10.09 | 2.33 | 2.11 | 0.50 | 4.79 | 1.58 |
| 61 | 0.28 | 0.32 | 8.09 | 1.87 | 1.38 | 0.25 | 5.85 | 1.72 |
| 62 | -0.18 | 0.15 | 9.05 | 2.32 | 1.98 | 0.50 | 4.57 | 1.65 |
| 63 | 0.27 | 0.19 | 11.12 | 1.13 | 1.80 | 0.09 | 6.19 | 0.70 |
| 64 | 0.24 | 0.10 | 8.52 | 0.65 | 1.70 | 0.21 | 5.02 | 0.72 |
| 65 |  |  |  |  |  |  |  |  |
| 66 | 0.18 | 0.07 |  |  |  |  |  |  |
| 69 |  |  |  |  |  |  |  |  |
| 70 | 0.09 | 0.20 | 5.81 | 0.98 | 1.68 | 0.23 | 3.47 | 0.75 |
| 71 | 0.07 | 0.19 | 6.85 | 0.49 | 1.88 | 0.15 | 3.64 | 0.40 |
| 72 | -0.16 | 0.04 |  |  |  |  |  |  |
| 73 | 0.19 | 0.07 | 5.30 | 0.23 | 1.74 | 0.07 | 3.05 | 0.19 |
| 74 | 0.04 | 0.06 |  |  |  |  |  |  |
| 75 |  |  |  |  |  |  |  |  |
| 76 | 0.02 | 0.04 | 5.50 | 0.52 | 2.05 | 0.14 | 2.69 | 0.31 |
| 77 | -0.02 | 0.05 | 7.49 | 0.80 | 1.65 | 0.19 | 4.54 | 0.72 |
| 78 | -0.16 | 0.04 |  |  |  |  |  |  |
| 79 | 0.06 | 0.04 | 9.20 | 0.54 | 2.07 | 0.12 | 4.45 | 0.37 |
| 80 | 0.18 | 0.09 |  |  |  |  |  |  |
| 109 |  |  |  |  |  |  |  |  |
| 110 |  |  |  |  |  |  |  |  |
| 111 |  |  | 11.45 | 1.15 | 2.25 | 0.29 | 5.08 | 0.83 |
| 112 | 0.06 | 0.05 |  |  |  |  |  |  |
| 113 | 0.21 | 0.08 |  |  |  |  |  |  |
| 114 | 0.20 | 0.04 |  |  |  |  |  |  |
| 119 |  |  |  |  |  |  |  |  |
| 120 | -0.08 | 0.04 |  |  |  |  |  |  |
| 121 |  |  |  |  |  |  |  |  |
| 122 | -0.02 | 0.13 | 4.86 | 0.69 | 2.57 | 0.33 | 1.89 | 0.36 |
| **MEDIAN** | **0.02** |  | **5.38** |  | **1.80** |  | **2.86** |  |

Data collected for BEX3 in 3.6 M urea and Buffer A, at 13 ^o^C on a Bruker Avance III spectrometer operating at 600 MHz (14.1 T). The standard deviation is shown in the columns identified as SD. The median for each dataset is shown at the bottom. Res# identifies the residue number while NOE stands for heteronuclear ^15^N{^1^H}-NOE.
